# A prospective phase II trial exploring the association between tumor microenvironment biomarkers and clinical activity of ipilimumab in advanced melanoma

**DOI:** 10.1186/1479-5876-9-204

**Published:** 2011-11-28

**Authors:** Omid Hamid, Henrik Schmidt, Aviram Nissan, Laura Ridolfi, Steinar Aamdal, Johan Hansson, Michele Guida, David M Hyams, Henry Gómez, Lars Bastholt, Scott D Chasalow, David Berman

**Affiliations:** 1 Melanoma Center, The Angeles Clinic and Research Institute, (2001 Santa Monica Blvd), Santa Monica, (90404), USA; 2 Department of Clinical Medicine and Department of Oncology, Aarhus University Hospital, (Nørrebrogade 44), Aarhus C, (DK-8000), Denmark; 3 Department of Surgery, Hadassah Hebrew University Medical Center, (P.O.Box 24035), Jerusalem, (il-91240), Israel; 4 Immunotherapy Unit, IRST Cancer Institute, (Via P. Maroncelli 40) Meldola, (47014), Italy; 5 Department of Oncology, Section for Clinical Cancer Research, Oslo University Hospital, (Ullernchausseen 70, OUS Radiumhospitalet), Oslo, (0310), Norway; 6 Department of Oncology-Pathology, Karolinska Institutet and Karolinska University Hospital Solna, (SE-171), Stockholm, (77), Sweden; 7 Department of Medical Oncology, National Institute of Cancer, (Viale Orazio Flacco 65), Bari, (70124), Italy; 8 Desert Surgical Oncology, (39000 Bob Hope Drive), Rancho Mirage, (92270), USA; 9 Department of Medicine, Instituto Nacional de Enfermedades Neoplásicas, (Avenida Angamos, Este 2520), Lima (34), Perú; 10 EORTC Melanoma Group, Odense University Hospital, (Sdr. Boulevard 29), Odense, (DK-5000), Denmark; 11 Exploratory Development, Global Biometric Sciences, Bristol-Myers Squibb Company, (Route 206 & Province Line Rd), Princeton, (08543), USA; 12 Research and Development, Discovery Medicine, Bristol-Myers Squibb Company, (Route 206 & Province Line Rd), Princeton, (08543), USA

**Keywords:** Cytotoxic T-lymphocyte antigen-4, FoxP3, indoleamine 2,3-dioxygenase, ipilimumab, melanoma, tumor biomarker, tumor-infiltrating lymphocytes

## Abstract

**Background:**

Ipilimumab, a fully human monoclonal antibody that blocks cytotoxic T-lymphocyte antigen-4, has demonstrated an improvement in overall survival in two phase III trials of patients with advanced melanoma. The primary objective of the current trial was to prospectively explore candidate biomarkers from the tumor microenvironment for associations with clinical response to ipilimumab.

**Methods:**

In this randomized, double-blind, phase II biomarker study (ClinicalTrials.gov NCT00261365), 82 pretreated or treatment-naïve patients with unresectable stage III/IV melanoma were induced with 3 or 10 mg/kg ipilimumab every 3 weeks for 4 doses; at Week 24, patients could receive maintenance doses every 12 weeks. Efficacy was evaluated per modified World Health Organization response criteria and safety was assessed continuously. Candidate biomarkers were evaluated in tumor biopsies collected pretreatment and 24 to 72 hours after the second ipilimumab dose. Polymorphisms in immune-related genes were also evaluated.

**Results:**

Objective response rate, response patterns, and safety were consistent with previous trials of ipilimumab in melanoma. No associations between genetic polymorphisms and clinical activity were observed. Immunohistochemistry and histology on tumor biopsies revealed significant associations between clinical activity and high baseline expression of FoxP3 (p = 0.014) and indoleamine 2,3-dioxygenase (p = 0.012), and between clinical activity and increase in tumor-infiltrating lymphocytes (TILs) between baseline and 3 weeks after start of treatment (p = 0.005). Microarray analysis of mRNA from tumor samples taken pretreatment and post-treatment demonstrated significant increases in expression of several immune-related genes, and decreases in expression of genes implicated in cancer and melanoma.

**Conclusions:**

Baseline expression of immune-related tumor biomarkers and a post-treatment increase in TILs may be positively associated with ipilimumab clinical activity. The observed pharmacodynamic changes in gene expression warrant further analysis to determine whether treatment-emergent changes in gene expression may be associated with clinical efficacy. Further studies are required to determine the predictive value of these and other potential biomarkers associated with clinical response to ipilimumab.

## Background

Historically, the prognosis for patients with stage IV metastatic melanoma has been very poor, with a median overall survival (OS) of 6-10 months and a 1-year survival rate of ~25% [1,2]. After decades of disappointments in the search for a melanoma therapy that could extend OS beyond that seen for the standard of care, two phase III randomized controlled trials demonstrated a statistically significant improvement in OS with ipilimumab in combination with dacarbazine in treatment-naïve patients [3] and as monotherapy in previously treated patients [4] with advanced melanoma. Ipilimumab is a fully human, monoclonal antibody that blocks cytotoxic T-lymphocyte antigen-4 (CTLA-4) [5,6], a molecule that down-regulates pathways of T-cell activation [7]. By blocking CTLA-4, ipilimumab potentiates an antitumor immune response [8-10].

Unlike conventional cancer therapies, the antitumor responses with ipilimumab may follow an initial period of apparent disease progression [11]. Ipilimumab therapy is associated with mechanism-based, immune-related adverse events (irAEs) that are inflammatory in nature [12], most of which are reversible using treatment guidelines provided in the US prescribing information [13]. However, treatment-related irAEs can be severe and, in rare instances, can be life-threatening [12]. Therefore, the ability to differentiate responders from nonresponders prior to initiation of therapy would help to maximize benefits associated with ipilimumab and minimize potential risks.

Biomarkers, intrinsic patient or tumor characteristics associated with clinical activity and/or toxicity of a given therapy, are increasingly demonstrating value towards the goals of personalized medicine. By allowing the prediction of both beneficial and detrimental responses to a given agent [14], biomarkers such as *HER-2 *in breast cancer [15] and *KRAS *in colon cancer [16] are beginning to change treatment paradigms. Promising biomarkers for melanoma include *KIT *and *BRAF *gene mutations [17,18].

Biomarkers of clinical efficacy for immunotherapies to treat melanoma are in early stages of development. A few studies of melanoma patients treated with immunotherapies have demonstrated association between clinical efficacy and gene expression profiles derived from tumor tissue [19]. These gene expression signatures featured many immune-related genes, indoleamine 2,3-dioxygenase (IDO), and FoxP3. The immunotherapies tested in these studies include high- dose interleukin (IL)-2, IL-12, peptide vaccines (including MAGE-A3), and dendritic cells pulsed with peptide vaccines [19]. Due to the fact that only a small number of patients had a response to therapy, there were few data points on which to base conclusions regarding associations between gene expression and efficacy. Previous studies of anti-CTLA-4 therapy in melanoma patients have shown an association between clinical activity and treatment-emergent changes in immune cells, such as increased absolute lymphocyte count and changes in specific T-cell populations (e.g., increased frequency of CD8+ cells) [20-24]. The primary objective of this exploratory, phase II trial (CA184-004; http://www.clinicaltrials.gov NCT00261365) was the prospective exploration of candidate biomarkers of clinical response to ipilimumab in the tumor microenvironment.

## Methods

### Patient population and study design

Previously treated and treatment-naïve patients with advanced melanoma were treated intravenously with 3 or 10 mg/kg ipilimumab every 3 weeks (Q3W) × 4 induction doses. At Week 24 (W24), eligible patients could receive ipilimumab maintenance treatment every 12 weeks (Q12W) or enter a companion study for follow-up and/or extended therapy.

**Figure 1:**
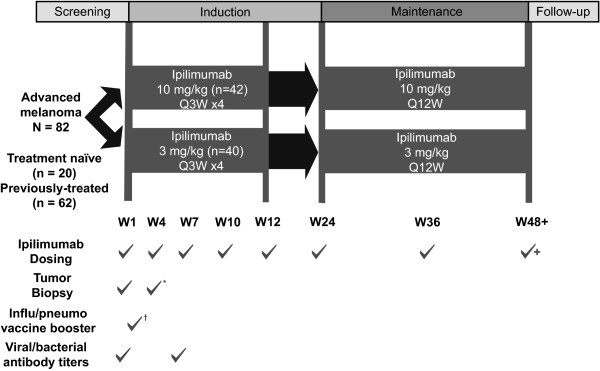
**Dosing and testing schedule: CA184-004**. *Tumor biopsy was performed at baseline and 24 to 72 hours after the second dose of ipilimumab. ^†^Influenza/pneumococcal booster administered 5 days after first dose of ipilimumab. Q3W: every 3 weeks; Q12W: every 12 weeks.

**Figure 2:**
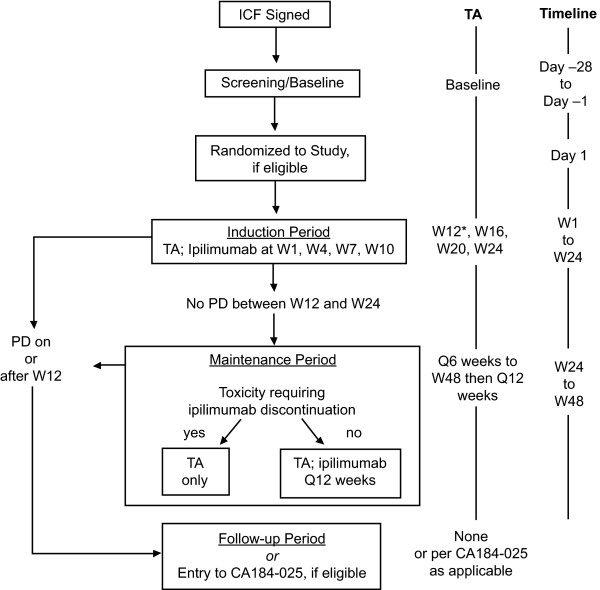
**CONSORT diagram: study CA184-004**. *Mandatory TA. CONSORT: Consolidated Standards of Reporting Trials; ICF: informed consent form; PD: progressive disease; TA: tumor assessment; W: study week.

The study design is outlined in Figures 1 and 2 (CONSORT diagram). Patients were of either gender, ≥ 18 years of age, and met the following main criteria: life expectancy ≥ 4 months; Eastern Cooperative Oncology Group performance status of 0-1; histologic or cytologic diagnosis of stage IV or unresectable stage III malignant melanoma; and measurable lesions. Pregnant women and individuals with ocular melanoma, serious autoimmune diseases, untreated active central nervous system (CNS) metastasis, and concomitant treatment with any anticancer or immunosuppressive agent were excluded.

The study was conducted in accordance with the Declaration of Helsinki, the International Conference on Harmonization Good Clinical Practice, the European Union Directive 2001/20/EC, and the United States Code of Federal Regulations 21CFR50. The protocol and patient-informed consent form received approval by Institutional Review Boards or Independent Ethics Committees. Written informed consent was obtained for all patients.

### Measurements of clinical efficacy

Investigator assessment of best overall response (BOR), determined between the date of first ipilimumab dose and the last tumor assessment (TA), was image-based and scored using modified World Health Organization criteria [25]. Patients who underwent excision or resection of any index lesions or received anticancer therapies other than ipilimumab were excluded from response evaluations.

For BOR, complete response (CR) required confirmation by a repeat, consecutive TA no less than 4 weeks after the criteria were first met; partial response (PR) required a similar, but not necessarily consecutive, repeat assessment; and stable disease (SD) included those who met criteria for SD at Week 12 and those with unconfirmed CR or PR. Efficacy endpoints included BOR rate (BORR) (total patients with CR or PR divided by total treated patients); disease control rate (DCR) (total patients with BOR of CR, PR, or SD divided by total treated patients); progression-free survival (PFS); OS; survival rate; response duration; proportion of patients with response duration ≥ 24 weeks; and time to response.

For the purpose of biomarker analyses, clinical activity was defined as a three-level categorical variable derived from BOR, with levels Benefit, Non-Benefit, and Unknown. The Benefit group included patients with BOR of CR, PR, or SD lasting ≥ 24 weeks from first ipilimumab dose. The Non-benefit group included patients with BOR of PD or SD ending prior to 24 weeks from first ipilimumab dose. The Unknown group included patients with unknown BOR, or BOR of SD with duration censored before 24 weeks from date of first dose and a death date either missing or at least 24 weeks from date of first dose.

### Safety assessments

Adverse events (AEs) were recorded based on MedDRA v10.0 system organ class (SOC) and Preferred Terms (PT) and listed by total AEs, serious AEs, AEs leading to ipilimumab discontinuation, and irAEs. Deaths within 70 days (5 ipilimumab half-lives) after last dose and cause of death were recorded. On-study and serious irAEs were summarized by SOC, PT, and worst grade, and analyzed for 5 subcategories: gastrointestinal, liver, skin, endocrine, and other.

### Biomarker assays

Tumor biopsies were performed prior to ipilimumab treatment and 24 to 72 hours after the second dose (W4). Fresh tumor biopsies were evaluated by a central, independent pathologist. Biopsies were evaluated by immunohistochemistry (IHC) for the candidate biomarkers FoxP3, granzyme B, perforin, CD4, CD8, CD45RO, and IDO; antibodies and conditions used for each analysis are detailed in Table S1 (Additional File 1). Four-micron serial sections of formalin-fixed, paraffin-embedded tissue blocks were mounted on glass microscope slides and dewaxed with four 5-minute changes of xylenes followed by a graded alcohol series to distilled water. Steam heat-induced epitope recovery (SHIER) was used with appropriate SHIER solution for 20 minutes in the capillary gap of the upper chamber of a Black and Decker^® ^Steamer [26]. Proteinase K digestion with and without SHIER pretreatment was also used. Slides were permanently cover-slipped with glass and CytoSeal and staining was assessed under a microscope.

Measures were scored on a 0 to 4 scale (in increments of 0.5 for 9 levels) using methods detailed in Table S2 (Additional File 2). Tumor characteristics (normal tissue, viable tumor, necrotic tumor, fibrotic regression, tumor-infiltrating lymphocytes [TILs], and peritumoral immune cells) were assessed by hematoxylin and eosin (H&E) staining; results were scored as 0%, ≤ 50%, or > 50% staining, also detailed in Table S2 (Additional File 2). TIL and peritumoral immune cell scores were based on percentage of the tumor; scores for the other characteristics were based on percentage of the entire specimen. IHC and H&E analyses were performed by an independent laboratory blinded to response status and timing of biopsy.

### Gene expression profiling using microarrays

Tumor biopsy messenger ribonucleic acid (mRNA) expression profiles were assessed using the HG-U133A_2 HT GeneChip™ microarray system (Affymetrix, Santa Clara, CA). Briefly, total RNA was extracted from fresh tissue samples using the Prism 6100 (Applied Biosystems, Foster City, CA), purified by RNAClean Kit (Agencourt Bioscience Corporation; Beverly, MA), and evaluated on a 2100 Bioanalyzer (Agilent Technologies, Santa Clara, CA). Complementary DNA preparation and hybridization on HT-HG-U133A 96-array plates followed manufacturer's protocols (Affymetrix, Santa Clara, CA). Appropriate Affymetrix control probe sets were examined to ensure quality control for the cDNA synthesis and the hybridization step.

### Genetic polymorphisms

Associations between genetic polymorphisms and clinical activity were investigated by genotyping peripheral blood for 20 single nucleotide polymorphisms (SNPs) and two deletions in 10 immune-related genes (BTNL2, CCR5, CD86, CTLA-4, IFNAR1, IFNAR2, IFNG, IL23R, NOD2, and PTPN22). The presence of allele *0201 at locus HLA-A and medium-resolution HLA-A and HLA-B genotypes were also assessed.

### Data analyses and statistical methodology

Efficacy analyses were based on all randomized patients or subsets of patients defined by baseline characteristics. BORR and frequencies of BOR values were calculated for each dose group. Exact 2-sided 95% confidence intervals (CIs) for within-dose-group BORR were calculated using the method of Clopper and Pearson [27]. Time to response in patients with BOR of CR or PR was summarized using descriptive statistics (median, minimum, maximum). PFS and OS within dose groups was estimated using the Kaplan-Meier product-limit method and included medians and corresponding 2-sided 95% CIs calculated using the method of Brookmeyer and Crowley [28]. One-year survival rate was calculated using the Kaplan-Meier product-limit method and reported with corresponding 2-sided 95% bootstrap CIs.

Response-evaluable patients in both dose groups were combined for biomarker analyses to increase the power to detect associations. Unless otherwise noted, all biomarker analyses were performed with S-PLUS 7.0.6 [29]. Using logistic regression of clinical activity on a biomarker, ignoring dose group, a 2-tailed likelihood-ratio test (LRT) of association with 80 patients and significance level of 0.05 has 90% power to detect an odds ratio (OR) of 2.24 (odds of activity when the biomarker is at its mean over that when it is 1 standard deviation from the mean).

Linear logistic regression was used to model the probability of clinical activity as an additive function of ipilimumab dose and pretreatment value of an IHC or H&E measure. IHC measures were used as continuous-valued predictors. H&E measures were used as binary predictors (positive score or not). *P*-values are from a logistic regression LRT of whether the effect of an IHC or H&E measure on probability of clinical activity is null. Linear logistic regression was used to model the probability of clinical activity as an additive function of ipilimumab dose and change from baseline of an IHC or H&E measure. *P*-values are from a logistic regression LRT of whether the effect of change from baseline is null.

Gene expression data were normalized using the Robust Multichip Average method [30] and the resulting log_2_-transformed expression levels were used for subsequent analyses. For each probe set, effects of time and ipilimumab dose on expression level were evaluated by fitting a linear mixed effects model with time, dose, and time-by-dose interactions as fixed effects and individual-patient overall mean expression level as random intercept. The fixed effects structure allows the model to estimate four different mean expression values, one at each time point in each dose group. The random intercept allows the model to account for a possible correlation between the pre- and post-treatment expression levels within each subject. Probe sets wherein expression changed over time were selected using a two-degrees-of-freedom F-test of the null hypothesis that there is no mean change from baseline (pretreatment) for either dose group. Probe sets wherein mean expression differed between dose groups were selected using a similar F-test of the null hypothesis that there is no difference in mean expression between dose groups for either time point. A false discovery rate (q-value) approach [31] was used to adjust for multiple testing. Analyses were performed using S-PLUS v7.0.6 for Linux, Bioconductor v1.14.0 (Affy package) [32] and q-value package v1.10.0 [29] for R v2.5.0 [33].

Pharmacodynamic analysis of gene expression was based on data from all treated patients with any such data; it was not limited to patients who had both pretreatment and post-treatment evaluable biopsies. Sixteen (16) patients had only pretreatment gene expression data. In theory, inclusion of these 16 patients may have introduced bias due to informative missingness. This bias is likely to be small, however, because (1) using a linear mixed effects model can reduce such bias; and (2) the clinical outcomes for the 16 patients having only pretreatment microarray data were similar to those for the 54 patients with both pre- and post-treatment microarray data (12.5% vs 11.0% CR or PR; 12.5% vs. 18.5% SD; and 75.0% vs. 70.5% PD or UN, respectively). An obvious alternative approach, complete-case analysis, would be subject to its own biases in the presence of informative missingness, whilst also suffering from reduced precision.

Exact tests of Hardy-Weinberg equilibrium were performed for gene polymorphisms, and logistic regression was used to model the probability of clinical activity as a function of genotype.

## Results

Fourteen sites in seven European, North American, and South American countries enrolled 101 patients; of these, one patient withdrew consent and 18 who no longer met study criteria were not randomized (12 due to CNS metastasis or active CNS disease, one due to stomach hemorrhage, one had no measurable disease, and four did not meet other inclusion/exclusion criteria). The remaining 82 patients were randomized between January 2006 and May 2007 to receive ipilimumab 3 mg/kg (n = 40) or 10 mg/kg (n = 42). Baseline characteristics of randomized patients (Table 1) were generally consistent between dose groups.

**Table 1:** Baseline characteristics - randomized patients.

Characteristic	Ipilimumab 3 mg/kg (n = 40)	Ipilimumab 10 mg/kg (n = 42)	Total (N = 82)
Male gender, %	70.0	57.1	63.4

Race, n (%)			
Asian	1 (2.5)	0	1 (1.2)
Black	0	1 (2.4)	1 (1.2)
White	39 (97.5)	41 (97.6)	80 (97.6)

Age in yrs, mean (range)	53.9 (23.0-78.0)	56.2 (26.0-87.0)	55.0 (23.0-87.0)
< 65, n (%)	31 (77.5)	29 (69.0)	60 (73.2)
≥ 65, n (%)	9 (22.5)	13 (31.0)	22 (26.8)

Disease stage, n (%)			
III	1 (2.5)	2 (4.8)	3 (3.7)
IV	39 (97.5)	40 (95.2)	79 (96.3)
M-stage, n (%)			
M0	0	2 (4.8)	2 (2.4)
M1A	12 (30.0)	9 (21.4)	21 (25.6)
M1B	6 (15.0)	3 (7.1)	9 (11.0)
M1C	22 (55.0)	28 (66.7)	50 (61.0)

ECOG-PS, n (%)			
0	25 (62.5)	27 (64.3)	52 (63.4)
1	15 (37.5)	15 (35.7)	30 (36.6)

Time since initial diagnosis in months, mean (range)	69.0 (1.1-384.0)	55.8 (3.8-237.9)	62.2 (1.1-384.0)

Any prior systemic therapy received, n (%)	29 (72.5)	33 (78.6)	62 (75.6)
Number received			
1, n (%)	15 (37.5)	19 (45.2)	34 (41.5)
2, n (%)	7 (17.5)	8 (19.0)	15 (18.3)
3, n (%)	6 (15.0)	5 (11.9)	11 (13.4)
≥ 5, n (%)	1 (2.5)	1 (2.4)	2 (2.4)

Any prior immunotherapy received, n (%)	(57.5)	(61.9)	
interleukin-2, n (%)	(45.0)	(38.1)	
interferon-α, n (%)	(22.5)	(19.0)	

Setting of prior therapy*			
Adjuvant therapy, n (%)	10 (25.0)	10 (23.8)	20 (24.4)
Metastatic disease, n (%)	26 (65.0)	28 (66.7)	54 (65.9)
Neoadjuvant therapy, n (%)	0	1 (2.4)	1 (1.2)

*Systemic therapy may have been received in > 1 setting.

ECOG-PS: Eastern Cooperative Oncology Group performance status.

### Clinical efficacy

There were no meaningful differences in clinical activity between the two ipilimumab doses (Table 2). During the study, patterns of tumor shrinkage after PD or mixed shrinkage and progression were consistent with those seen in other ipilimumab melanoma trials [34-36]. Two patients with BOR of PD were followed beyond PD without administration of other anticancer treatments; one patient in the 10 mg/kg group had subsequent shrinkage and disappearance of lesions and one patient in the 3 mg/kg group had progression of an index lesion and shrinkage of others. Across groups, ongoing responses were observed in 7 of 8 responders at database lock. Due to the small number of patients in each dose group with BOR of CR, PR, or SD, only one of whom progressed on study (results not shown), results were inconclusive for response duration and disease control, major durable response rate (proportion of patients with CR or PR lasting ≥ 24 weeks), and major durable disease control rate (proportion of patients with CR, PR, or SD lasting ≥ 24 weeks). Forty-five percent of patients in the 3 mg/kg group and 47.6% in the 10 mg/kg group died on-study; median follow-up was 8.9 months and 8.6 months, respectively.

**Table 2:** Efficacy summary.

Efficacy Measure	Ipilimumab 3 mg/kg (n = 40)*	Ipilimumab 10 mg/kg (n = 42)*
Response, n (%)		
CR	0	1 (2.4)
PR	3 (7.5)	4 (9.5)
SD	10 (25.0)	3 (7.1)
PD	19 (47.5)	24 (57.1)
Unknown	8 (20.0)	10 (23.8)

BORR, % (95% CI)	7.5 (1.6, 20.4)	11.9 (4.0, 25.6)

DCR, %	32.5	19.0

OS, median, in months	12.9	11.8

1-year survival rate^†^, % (95% CI)	60.9 (41.7, 74.9)	44.2 (24.1, 64.1)

PFS, median (95% CI), in months	2.63 (2.56, 3.88)	2.56 (2.50, 2.66)

Progressed or died, n (%)	28 (70.0)	32 (76.2)

Time to response, median (min- max), in months	2.5 (2.1-5.3)	2.6 (2.6-3.6)

*Four patients (10.0%) in the 3 mg/kg group and five (11.9%) in the 10 mg/kg group were censored from the analyses of response-based endpoints (BORR, PFS, DCR, time to response) because of excision or resection of index lesions.

^†^Estimated.

BORR: best overall response rate; CI: confidence interval; CR: complete response; DCR: disease control rate; OS: overall survival; PD: progressive disease; PFS: progression-free survival; PR: partial response; SD: stable disease.

### Safety

Safety results are summarized in Table 3. The incidence of irAEs was similar between dose groups; however, irAEs were generally less severe with the lower dose. Two drug-related deaths due to large intestine perforation were reported: one in the 3 mg/kg group within 70 days of last ipilimumab dose and one in the 10 mg/kg group more than 70 days after last ipilimumab dose.

**Table 3:** Safety summary.

Event	Ipilimumab 3 mg/kg (n = 40)	Ipilimumab 10 mg/kg (n = 42)
**Death**, n (%)		
All	18 (45.0)	20 (47.6)
Within 70 days of last dose	8 (20.0)	8 (19.0)
Within 30 days of last dose	3 (7.5)	4 (9.5)

**Serious AE**, n (%)		
All	18 (45.0)	20 (47.6)
Drug-related	7 (17.5)	8 (19.0)

**AE leading to study discontinuation**, n (%)	5 (12.5)	11 (26.2)

**Drug-related AE**, n (%)		
Any grade	33 (82.5)	32 (76.2)
Grade 3-4	6 (15.0)	13 (31.0)
Grade 5	1 (2.5)	1 (2.4)

**irAE**, n (%)		
***Overall***		
Any grade	22 (55.0)	28 (66.7)
Grade 3-4	3 (7.5)	8 (19.0)
Grade 5	1 (2.5)	1 (2.4)

***GI***		
Any grade	11 (27.5)	19 (45.2)
Grade 3-4	3 (7.5)	3 (7.2)
Grade 5	1 (2.5)	1 (2.4)

***Liver***		
Any grade	0	2 (4.8)
Grade 3-4	0	2 (4.8)

***Endocrine***		
Any grade	2 (5.0)	2 (4.8)
Grade 3-4	0	2 (4.8)

***Skin***		
Any grade	16 (40.0)	21 (50.0)
Grade 3-4	0	1 (2.4)

***Other***		
Any grade	1 (2.5)	3 (7.1)
Grade 3-4	0	1 (2.4)

AE: adverse event; GI: gastrointestinal; irAE: immune-related adverse event.

### Biomarker Evaluation - Primary Objective

To assess potential associations between clinical activity and putative biomarkers, patients were classified into three groups based on BOR (see Methods subsection *Measurements of clinical efficacy*). The Benefit group included patients with BOR of CR, PR, or SD lasting ≥ 24 weeks from first ipilimumab dose. Of the 82 patients treated in CA184-004, 12 patients (14.6%) were in the Benefit Group, 50 (61.0%) were in the Non-benefit Group, and 20 (24.4%) were in the Unknown category.

**Figure 3:**
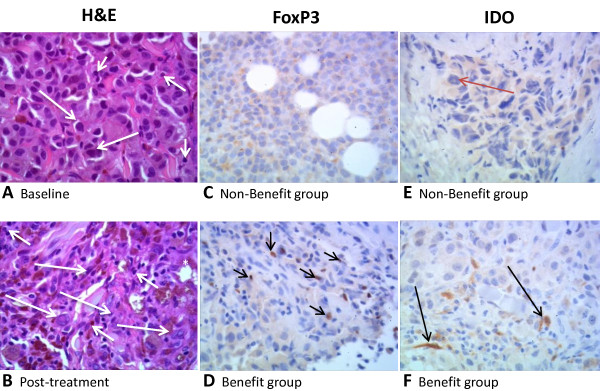
**Tumor tissue samples from patients with malignant melanoma treated with ipilimumab**. 60× images of skin (A, B, F, Subject 04055; E, Subject 04024) and soft tissue (C, Subject 04066; D, Subject 04002) involved with metastasis and infiltration of melanoma cells, respectively. (A-B) Skin under the epidermis stained with hematoxylin and eosin (H&E) before (A) and after (B) treatment with ipilimumab. Melanoma cells are characterized by abundant cytoplasm, large and central nuclei, apparent nucleolus (large arrows). In contrast, melanin pigment (star) is associated with mononuclear leukocytes (small arrows). Note the increase in tumor-infiltrating mononuclear leukocytes (TILs) post-treatment (B) relative to the baseline (A) in this clinical benefit subject. (C-D) FoxP3 positive staining (with anti-FOXP3) of the nuclei of mononuclear leukocytes (small arrows) in a clinical benefit subject at baseline (D). Non-clinical benefit subject (C) shows no staining of mononuclear leukocytes at baseline. (E-F) IDO expression (anti-IDO staining) at baseline in a clinical benefit subject (F) shows staining of mononuclear leukocytes (small arrows), and spindloid and endothelial cells (large arrows). IDO expression is minimal in the non-clinical benefit subject (E) at baseline, showing focal and weak staining of melanoma cells (red arrow). H&E scores for tumor-associated infiltrating mononuclear leukocytes: (A) ≤ 50%, (B) > 50%. Staining scores for FoxP3: (C) 0, (D) 1. Staining scores for IDO: (E) 0, (F) 1.

Ninety-one tumor biopsies (50 pretreatment, 41 post-treatment) from 57 patients were sectioned and stained using hematoxylin and eosin (H&E) to visualize tumor infiltrating lymphocytes (TILs) (Figure 3A, B), or using immunohistochemistry (IHC) to evaluate other biomarkers (Figure 3C-F). The number of samples evaluable varied among individual biomarkers examined, as indicated in the tables summarizing these results. To increase the power of the study, biomarker results from both dose groups were pooled.

H&E-stained sections were scored to quantify the prevalence of TILs (Additional File 2 Table S2). Although there was not convincing evidence that baseline TIL scores were associated with clinical activity, a statistically significant (p = .005) association was observed between change from baseline in TILs and clinical activity (Table 4, Additional File 3 Table S3, Figure 3A and 3B). In the Benefit group, 57.1% of patients had a post-treatment increase in TILs and none had a decrease. In contrast, 10.0% of Non-benefit group patients had an increase in TILs relative to baseline and 15.0% had a decrease. The estimated OR of 13.27 indicates that the odds of benefit increased approximately 13-fold for each one-unit increase from baseline in TIL score.

**Table 4:** Association of clinical activity with tumor biomarkers.

Biomarker	Observed Effect		*P-*value*
		
	Benefit Group (n = 21)	Non-benefit Group (n = 43)	
TILs change from baseline to week 4, by H&E	4/7 increase 0/7 decrease 3/7 no change	2/20 increase 3/20 decrease 15/20 no change	0.005

FoxP3 expression at baseline, by IHC	6/8 evaluable patients positive	9/25 evaluable patients positive	0.014

IDO expression at baseline, by IHC	3/8 evaluable patients positive	3/27 evaluable patients positive	0.012

*P-values are for tests of association between biomarker and clinical activity and are uncorrected for multiple testing.

H&E: hematoxylin and eosin; IDO: indoleamine 2,3-dioxygenase; IHC: immunohistochemistry; TILs: tumor-infiltrating lymphocytes.

Figure 3 shows examples of FoxP3 (C, D) and IDO (E, F) immunostaining. At baseline, FoxP3 staining was apparent in the nuclei of mononuclear leukocytes, and was more prominent in samples from patients that later derived clinical benefit from ipilimumab treatment (D) than in samples from the Non-benefit group (C). Likewise, pretreatment samples showed stronger IDO staining for patients that later derived clinical benefit from treatment (F) than those who did not (E).

IHC-stained sections were scored (as described in Additional File 2 Table S2) to facilitate statistical analysis of the trends observed. Statistically significant associations were observed between clinical activity and pretreatment FoxP3 and IDO expression (p = 0.014 and 0.012, respectively) (Table 4, Additional Files 4 and 5 Tables S4 and S5). FoxP3 was detected in 75.0% of evaluable pretreatment biopsies in the Benefit group and 36.0% in the Non-benefit group. IDO was detected in 37.5% of evaluable pretreatment biopsies in the Benefit group and 11.1% in the Non-benefit group. Estimated ORs for FoxP3 and IDO were 10.38 and 8.72, respectively, indicating that the odds of benefit increased approximately 10- and 9-fold, respectively, for each one-unit increase in pretreatment score. No associations were apparent between clinical activity and total infiltrate; expression of CD4, CD45RO, CD8, granzyme B, or perforin; or amount of normal tissue, viable tumor, necrotic tumor, fibrotic regression, or peritumoral immune cells.

### Pharmacodynamic effects on gene expression

mRNA expression profiles were measured in 70 pretreatment (baseline) and 58 post-treatment (W4) fresh tumor biopsy samples (one replicate per sample); 54 represented matched pairs from the same patient. Although not reported, reasons for lack of evaluable biopsy may include poor sampling technique or insufficient tissue. Of 22,215 initial probe sets, 17,519 with maximum log_2 _expression level > 4 and coefficient of variation > 5% across all samples were analyzed further. These filters remove probe sets with very low expression levels in all samples or with very little variability across all samples, respectively. They were applied because unexpressed probe sets or probe sets with nearly constant expression were not of interest. After correcting for multiple testing, 466 probe sets demonstrated significant (q-value < 0.05) changes from baseline (Additional File 6 Figure S1): 286 had estimated increases in expression from baseline for both the 3 and 10 mg/kg dose groups (Additional File 7 Table S6); 165 had estimated decreases in expression from baseline for both dose groups (Additional File 8 Table S7); and 15 had an opposite direction of estimated change from baseline for the two dose groups (Additional File 9 Table S8).

Significant increases in post-treatment expression from baseline were seen for various immune-response genes (immunoglobulins, granzyme B, perforin-1, granulysin, CD8 β-subunit, and T-cell receptor-α and -β subunits). Genes with significantly decreased expression from baseline included at least one known melanoma antigen: tyrosinase-related protein-2 (DCT). Of all the genes tested, DCT had the strongest negative time effect at 3 mg/kg, followed by solute carrier family 45, member 2 (a protein that is present in a high percentage of melanoma cell lines), G protein-coupled receptor 143, and carbonic anyhydrase XIV. After multiplicity correction, no probe sets met the q-value threshold of < 0.05 for significant dose effect or demonstrated a significant difference between dosage groups in mean change in expression from baseline using a one-degree-of-freedom F-test of the time-by-dose interaction.

### Genetic polymorphisms did not predict clinical activity

Allele and genotype frequencies for 22 genetic polymorphisms in peripheral blood were summarized for all response-evaluable patients (Table 5). Of the 22 polymorphisms, 20 were SNPs and 2 (NOD2 rs5743293 and CCR5 CCR5d32 [rs333]) were deletions; the term SNPs will be used to refer to all of the polymorphisms. A genotype score for at least one SNP was available for 76 treated patients of whom 65 were response evaluable. All but two patients with SNP data were Caucasian, so analyses were not stratified by race. No SNPs were monomorphic in this sample of patients. No statistically significant departures from Hardy-Weinberg equilibrium were observed. For SNPs rs2066844, rs2066845, and rs5743293 in gene NOD2, only 2 to 4 patients possessed a minor allele. Because of such limited polymorphism, these SNPs were excluded from further analyses. For the remaining 19 SNPs, no statistically significant associations with clinical activity were observed (Table 6); however, there was limited power to detect such associations given the relatively small number of patients with SNP data.

**Table 5:** Allele and genotype distributions for 22 genetic polymorphisms: all response-evaluable patients with genotype data.

Gene	Poly-morphism	Major Allele (1)	Minor Allele (2)	HWE *P*-value	Minor Allele Frequency (%)	Number of Patients (%)				
						
						Genotype 11	Genotype 12	Genotype 22	Genotype Unknown	Total
BTNL2	rs2076530	A	G	0.31	41.4	24 (37.5)	27 (42.2)	13 (20.3)	1 (1.5)	65 (85.5)

CCR5	CCR5d32	wt	d32	0.49	10.0	53 (81.5)	11 (16.9)	1 (1.5)	0 (0.0)	65 (85.5)

CCR5	rs1799987	A	G	0.30	39.2	26 (40.0)	27 (41.5)	12 (18.5)	0 (0.0)	65 (85.5)

CD86	rs1129055	G	A	0.15	31.5	33 (50.8)	23 (35.4)	9 (13.8)	0 (0.0)	65 (85.5)

CD86	rs2681417	A	G	1.00	2.4	60 (95.2)	3 (4.8)	0 (0.0)	2 (3.1)	65 (85.5)

CTLA4	rs11571317	C	T	1.00	4.6	59 (90.8)	6 (9.2)	0 (0.0)	0 (0.0)	65 (85.5)

CTLA4	rs1863800	C	T	0.79	37.3	24 (38.1)	31 (49.2)	8 (12.7)	2 (3.1)	65 (85.5)

CTLA4	rs231775	A	G	0.19	39.8	26 (40.6)	25 (39.1)	13 (20.3)	1 (1.5)	65 (85.5)

CTLA4	rs3087243	G	A	1.00	36.5	25 (39.7)	30 (47.6)	8 (12.7)	2 (3.1)	65 (85.5)

CTLA4	rs4553808	A	G	0.30	23.4	39 (60.9)	20 (31.2)	5 (7.8)	1 (1.5)	65 (85.5)

IFNAR1	rs2257167	G	C	0.75	26.2	36 (55.4)	24 (36.9)	5 (7.7)	0 (0.0)	65 (85.5)

IFNAR2	rs7279064	T	G	0.01	34.6	23 (35.4)	39 (60.0)	3 (4.6)	0 (0.0)	65 (85.5)

IFNG	rs2069705	T	C	0.58	34.6	29 (44.6)	27 (41.5)	9 (13.8)	0 (0.0)	65 (85.5)

IFNG	rs2430561	A	T	0.80	46.9	17 (26.6)	34 (53.1)	13 (20.3)	1 (1.5)	65 (85.5)

IL23R	rs1004819	C	T	0.44	38.5	26 (40.0)	28 (43.1)	11 (16.9)	0 (0.0)	65 (85.5)

IL23R	rs11209026	G	A	1.00	7.7	55 (84.6)	10 (15.4)	0 (0.0)	0 (0.0)	65 (85.5)

IL23R	rs2201841	T	C	0.59	35.9	25 (39.1)	32 (50.0)	7 (10.9)	1 (1.5)	65 (85.5)

IL23R	rs7517847	T	G	0.43	39.8	25 (39.1)	27 (42.2)	12 (18.8)	1 (1.5)	65 (85.5)

NOD2	rs2066844	C	T	1.00	1.5	63 (96.9)	2 (3.1)	0 (0.0)	0 (0.0)	65 (85.5)

NOD2	rs2066845	G	C	1.00	1.6	62 (96.9)	2 (3.1)	0 (0.0)	1 (1.5)	65 (85.5)

NOD2	rs5743293	D	I	1.00	2.3	62 (95.4)	3 (4.6)	0 (0.0)	0 (0.0)	65 (85.5)

PTPN22	rs2476601	G	A	1.00	4.6	59 (90.8)	6 (9.2)	0 (0.0)	0 (0.0)	65 (85.5)

11 = major-allele homozygote; 12 = heterozygote; 22 = minor-allele homozygote.

Denominator for Minor Allele Percent is number of known alleles in row.

Denominator for percents in columns "11", "12", and "22" is number of patients in row who have a known genotype.

Denominator for percents in column "Genotype Unknown" is total number of patients in row.

Denominator for percents in column "Total" is total number of patients with genotype data.

rs1799987 is also known as rs28897671; rs2430561 is also known as (+ 874A- > T); CCR5d32 = rs333.

**Table 6:** Prediction of clinical activity status by genotype at each of 19 genetic polymorphisms: all response-evaluable patients with genetic polymorphism data and status = benefit or non-benefit.

Gene	Polymorphism	N	DF	*P*-value	OR: Genotype 1/2 vs 1/1		OR: Genotype 2/2 vs 1/1	
					Estimate	95% CI	Estimate	95% CI
BTNL2	rs2076530	56	2	0.48	0.40	0.08, 1.93	0.81	0.12, 5.25

CCR5	CCR5d32	57	2	0.54	0.41	0.04, 3.83	0.00	0.00, Inf

CCR5	rs1799987	57	2	0.80	0.76	0.17, 3.39	1.35	0.25, 7.35

CD86	rs1129055	57	2	0.11	1.92	0.50, 7.38	0.00	0.00, Inf

CD86	rs2681417	55	1	0.52	2.39	0.18, 31.0	NA	NA, NA

CTLA4	rs11571317	57	1	0.78	0.73	0.07, 7.25	NA	NA, NA

CTLA4	rs1863800	55	2	0.06	0.35	0.08, 1.45	0.00	0.00, Inf

CTLA4	rs231775	56	2	0.05	0.43	0.07, 2.73	3.75	0.72, 19.42

CTLA4	rs3087243	55	2	0.07	0.39	0.09, 1.62	0.00	0.00, Inf

CTLA4	rs4553808	56	2	0.08	0.19	0.02, 1.76	3.25	0.37, 28.28

IFNAR1	rs2257167	57	2	0.98	0.93	0.23, 3.80	1.24	0.10, 14.60

IFNAR2	rs7279064	57	2	0.59	0.83	0.22, 3.15	0.00	0.00, Inf

IFNG	rs2069705	57	2	0.79	0.71	0.18, 2.82	0.50	0.05, 5.31

IFNG	rs2430561	56	2	0.67	1.22	0.20, 7.31	2.36	0.30, 18.72

IL23R	rs1004819	57	2	0.95	0.81	0.19, 3.37	0.97	0.14, 6.52

IL23R	rs11209026	57	1	0.40	0.42	0.05, 3.93	NA	NA, NA

IL23R	rs2201841	56	2	0.82	0.64	0.15, 2.65	0.80	0.07, 9.43

IL23R	rs7517847	56	2	0.35	2.33	0.50, 10.85	0.67	0.06, 7.77

PTPN22	rs2476601	57	1	0.95	0.93	0.09, 9.69	NA	NA, NA

1/1 = major-allele homozygote; 1/2 = heterozygote; 2/2 = minor-allele homozygote.

CI = Confidence Interval.

DF = degrees of freedom for likelihood-ratio test (LRT).

Inf = infinite.

N = number of patients with non-missing genotypes.

OR = odds ratio comparing the odds of Status = Benefit between two genotype classes. OR > 1 means that the odds of Benefit were greater for class 1/2 or 2/2 than for class 1/1.

NA = not estimable.

*P*-value is for a logistic regression LRT of whether both (if > 1 estimable) odds ratios = 1.

Presence or absence of allele HLA-A*0201 could be determined for 67 of the 71 response-evaluable patients. Medium-resolution HLA genotypes also could be obtained for 67 of these patients, but presence/absence status of the five HLA alleles present in at least 10% of patients could not be determined for 5 to 15 of these patients. Presence of allele *0201 at locus HLA-A was cross-tabulated by clinical activity status (Table 7). No association between presence of this allele and clinical activity was apparent. Similarly, presence of the five common HLA-A alleles was cross-tabulated by clinical activity status (Table 8) and no associations were apparent.

**Table 7:** Presence of allele *0201 at locus HLA-A by clinical activity status: all response-evaluable patients.

Clinical Activity Status	n (%)			
	
	Allele Absent	Allele Present	Allele Status Unknown	Total
Benefit	4 (33.3)	8 (66.7)	0 (0.0)	12 (16.9)
CR or PR	3 (37.5)	5 (62.5)	0 (0.0)	8 (11.3)
Prolonged SD	1 (25.0)	3 (75.0)	0 (0.0)	4 (5.6)

Non-benefit	21 (45.7)	25 (54.3)	3 (6.1)	49 (69.0)

Unknown	6 (66.7)	3 (33.3)	1 (10.0)	10 (14.1)

Total	31 (46.3)	36 (53.7)	4 (5.6)	71 (100.0)

Denominator for percents in columns "Allele Absent" and "Allele Present" is number of patients in row who have a known allele status.

Denominator for percents in column "Allele Status Unknown" is total number of patients in row.

Denominator for percents in column "Total" is number of response-evaluable patients.

**Table 8:** Medium-resolution HLA-A genotyping: status of common alleles by clinical activity status: all response-evaluable patients with medium-resolution HLA genotype data.

Allele*	Clinical Activity Status	n (%)			
		
		Allele Present	Allele Absent	Allele status unknown	Total
02	Benefit	8 (72.7)	3 (27.3)	1 (8.3)	12 (17.9)
	Non-Benefit	28 (65.1)	15 (34.9)	3 (6.5)	46 (68.7)
	Unknown	3 (37.5)	5 (62.5)	1 (11.1)	9 (13.4)
24	Benefit	1 (10.0)	9 (90.0)	2 (16.7)	12 (17.9)
	Non-Benefit	12 (28.6)	30 (71.4)	4 (8.7)	46 (68.7)
	Unknown	2 (25.0)	6 (75.0)	1 (11.1)	9 (13.4)
03	Benefit	2 (20.0)	8 (80.0)	2 (16.7)	12 (17.9)
	Non-Benefit	9 (21.4)	33 (78.6)	4 (8.7)	46 (68.7)
	Unknown	2 (25.0)	6 (75.0)	1 (11.1)	9 (13.4)
01	Benefit	1 (16.7)	5 (83.3)	6 (50.0)	12 (17.9)
	Non-Benefit	8 (20.5)	31 (79.5)	7 (15.2)	46 (68.7)
	Unknown	2 (28.6)	5 (71.4)	2 (22.2)	9 (13.4)
11	Benefit	2 (20.0)	8 (80.0)	2 (16.7)	12 (17.9)
	Non-Benefit	6 (14.3)	36 (85.7)	4 (8.7)	46 (68.7)
	Unknown	1 (12.5)	7 (87.5)	1 (11.1)	9 (13.4)

*Only alleles present in at least 10% of all patients are shown.

Denominator for percents in columns "Allele Present" and "Allele Absent" is number of patients in row with known allele status.

Denominator for percents in column "Allele Status Unknown" is row total.

Denominator for percents in column "Total" is 67, the total number of patients in the data set.

## Discussion

Clinical activity and safety results from the present study are similar to those of other clinical trials of ipilimumab in advanced melanoma. In this study, BORRs of 7.5% and 11.9% and DCRs of 32.5% and 19% were observed for the 3 mg/kg and 10 mg/kg groups, respectively. There results are comparable to BORRs of 5.8% to 15.8% and DCRs of 20% to 30% for 10 mg/kg ipilimumab monotherapy in phase II studies [8-10], and to a BORR of 10.9% and DCR of 28.5% for 3 mg/kg ipilimumab monotherapy in a phase III trial [4]. The 1-year survival rates of 60.9% and 44.2% for 3 and 10 mg/kg ipilimumab, respectively, were also similar to those of previous phase II studies (47.2% to 62.4% for 10 mg/kg ipilimumab) [8-10] and to the phase III trial (45.6% for 3 mg/kg ipilimumab) [4]. Safety results from CA184-004 are consistent with those from a combined analysis of 325 patients in phase II trials of 10 mg/kg ipilimumab [37]. In the 10 mg/kg arm, drug-related AEs and irAEs occurred in 76.2% and 66.7% of patients, respectively, compared with 84.6% and 72.3% from the pooled analysis. As in previous studies, mechanism-based irAEs affecting the skin and gastrointestinal system were the most common toxicities observed and irAEs were generally reversible using product-specific treatment guidelines [4].

Variations within an individual patient's genome or somatic changes in the tumor DNA can affect disease progression and management. An increased understanding of how genetic variation can influence disease processes may allow physicians to personalize medicine through the design of individualized strategies for monitoring, prevention, and treatment that are the most effective for a given patient. Determination of a patient's genetic profile may contribute to the primary goals of personalized medicine by guiding the selection of treatments that maximize the chance for successful outcomes while minimizing adverse events [38]. Interest in personalized medicine is growing within the oncology community as evidence accumulates showing that differential clinical responses to a number of anticancer drugs can be associated with characteristics of an individual patient.

Recent reports include examples of this from studies of both melanoma and other cancers. Vemurafenib (PLX4032) is a selective inhibitor of the oncogenic V600E mutant BRAF kinase. In a melanoma study of 21 patients who received a sufficiently high dose of vemurafenib, 5 without the V600E BRAF mutation did not respond to the drug; however, of 16 patients with the mutation, 9 responded to vemurafenib and 7 did not [18]. In a phase II study of imatinib for advanced melanoma, a substantial proportion of patients with tumors characterized by mutation or amplification of KIT responded to the drug whereas it had limited activity in a nonselected population of melanoma patients [39]. Results of the CRYSTAL trial indicate that the clinical activity of cetuximab against metastatic colorectal cancer is enhanced in patients with wild-type KRAS compared with individuals having KRAS mutations, none of whom benefited from cetuximab treatment [40]. The identification of predictive biomarkers for immunotherapies such as ipilimumab will likely require interrogation of the immune functional status of the patient in addition to genomic analysis.

The presence of TILs, indicating an antitumor immune response, is a favorable prognostic indicator in a number of cancers [41-45]; however, the prognostic significance of TILs in melanoma remains unclear [46]. In this study, CA184-004, the clinical activity of ipilimumab was positively associated with an increase in TILs from baseline, suggesting that the infiltrating lymphocytes may have anti-tumor activity, and that changes in the immune microenvironment of the tumor may slow disease progression. There likely are other changes in immune cell demographics, localization, and function that are involved in melanoma progression. Peripheral blood absolute lymphocyte count (ALC) has also demonstrated prognostic value in a number of malignancies [47-50]. Association between clinical activity of ipilimumab and change from baseline in ALC was studied in the current trial [24] and is being explored further.

In the current study, higher protein levels of IDO and FoxP3 at baseline were significantly positively associated with clinical outcome and therefore may be predictive of ipilimumab clinical activity. Increased expression of IDO has been associated with an immunosuppressive tumor microenvironment or a response to chronic inflammation [51,52]. In melanoma, baseline expression of IDO by tumors or infiltrates has been associated with faster tumor growth, lower antitumor T-cell responses, and poor prognosis [53]; however, recent data suggest that IDO may induce apoptosis of melanoma tumor cells [54]. Furthermore, the presence of IDO might be a consequence of an active immune microenvironment since IDO expression is induced by pro-inflammatory cytokines such as IL-1, interferon-γ and tumor necrosis factor-α [55-57]. It is possible that IDO expression is up-regulated in tumors from patients with an ongoing, albeit suboptimal, antitumor immune response and that these individuals may be more likely to respond to ipilimumab. Further investigation of the role of IDO in melanoma and its value as a biomarker for ipilimumab is warranted.

FoxP3 has been identified as a marker of natural and adaptive/induced regulatory T cells (T_regs_) [58]. Patients with cancer have higher levels of T_regs _in peripheral blood than healthy individuals [59], leading to a suppression of T-cell immunity that has an immunopathologic role in tumor growth [56] and influences prognosis in many cancers [60-63]. Metabolic degradation of tryptophan by IDO promotes the conversion of naïve T cells to T_regs _[64,65]. Thus, intratumoral expression of IDO may have promoted the accumulation of FoxP3^+ ^cells; however, recent findings suggest that FoxP3 may not be specific for T_regs_, but can also be expressed by other activated T-cell populations [66]. Other reports suggest that the ratio of effector T cells (T_effs_) to T_regs_, rather than changes in the absolute number of intratumoral T_effs _or T_regs_, may be a more specific biomarker of effective antitumor immune responses elicited by CTLA-4 blockade [67,68].

Results from CA184-004 are similar to those found with tremelimumab, another anti-CTLA-4 antibody. In a pilot study of 15 tumor biopsies from 7 tremelimumab-treated patients, clinical response was associated with increased tumor infiltration by CD8^+ ^TILs whereas a more variable association was found with tumor infiltration of CD4^+ ^TILs [69]. The presence of FoxP3^+ ^regulatory T cells, indicative of an immunosuppressive tumor microenvironment, was also variably associated with positive response. No apparent association between clinical response and intratumoral expression of IDO was seen in that study.

In addition to FoxP3 and IDO, other proteins likely are involved in creating an anti-tumor immune microenvironment. Pharmacodynamic microarray data on mRNA derived from tumor samples showed that ipilimumab led to altered gene expression in the tumor microenvironment, significantly increasing expression of immune-related genes, and significantly decreasing the expression of genes linked to melanoma. This analysis provides a list of candidate genes for further investigation to determine whether changes in gene expression support anti-tumor activity and/or are associated with clinical efficacy. Protein levels do not always correlate with mRNA expression and so candidate genes of interest should be confirmed by protein assays. Analyses of the relationship between expression changes and clinical efficacy are described in a separate manuscript that has been submitted for publication. In a previous study of ipilimumab in melanoma patients, associations between SNPs in the CTLA-4 gene and objective response were suggested [70]; such associations were not observed in the current study. Consistent with a previous study reporting that ipilimumab-treated patients with advanced melanoma have similar survival outcomes regardless of their HLA-A*0201 status [71], the current study found no association between clinical activity and the presence of the *0201 allele or any of the five most common HLA-A alleles.

The associations of clinical activity with increased TILs during treatment and with baseline expression of IDO and FoxP3 suggest that an immune-active tumor microenvironment might be necessary for a favorable response to ipilimumab. The increased expression of immune-related genes after treatment with ipilimumab suggest additional genes to be tested for their involvement in the creation of this immune-active tumor microenvironment.

## Conclusions

Baseline expression of immune-related tumor biomarkers and a post-treatment increase in TILs may be positively associated with ipilimumab clinical activity. These preliminary results are hypothesis generating and support the need for larger prospective trials to confirm these findings and further characterize other potential biomarkers for ipilimumab.

## Competing interests

OH, HS, AN, LR, SA, JH, MG, DMH, HG, and LB declare that they have no competing interests. DB and SDC are employees of Bristol-Myers Squibb.

## Authors' contributions

All authors read and approved the final manuscript. OH was the principal study investigator for clinical trial CA184-004, and participated in drafting and revising the manuscript. HS, AN, LR, SA, JH, MG, DMH, HG, and LB were study investigators for the CA184-004 trial and reviewed and provided comments during manuscript preparation. SDC participated in the design of the study, design and execution of statistical analyses, and drafting the manuscript. DB participated in the design and conduct of the study and drafting of the manuscript.

## Supplementary Material

Additional file 1 **Table S1**. Specifications for antibodies used in IHC analyses. Click here for file

Additional file 2 **Table S2**. Specifications Scoring of IHC and H&E-stained samples. Click here for file

Additional file 3 **Table S3**. Joint frequencies of clinical activity and change from baseline of tumor biopsy H&E scores: TILs. Click here for file

Additional file 4 **Table S4**. Joint frequencies of clinical activity and pretreatment tumor biopsy IHC scores: FoxP3. Click here for file

Additional file 5 **Table S5**. Joint frequencies of clinical activity and pretreatment tumor biopsy IHC scores: FoxP3. Joint frequencies of clinical activity and pretreatment tumor biopsy IHC scores: IDO. Click here for file

Additional file 6 **Figure S1**. mRNA Expression in tumor biopsies: scatter plot summarizing magnitude and significance of changes from baseline. Click here for file

Additional file 7 **Table S6**. Model estimates for probe sets with time effect q-value < 0.05 and expression level increased from baseline. Click here for file

Additional file 8 **Table S7**. Model estimates for probe sets with time effect q-value < 0.05 and expression level decreased from baseline. Click here for file

Additional file 9 **Table S8**. Model estimates for probe sets with time effect q-value < 0.05 and opposite direction of change from baseline. Click here for file
