# Supplementary material for: A prospective phase II trial exploring the association between tumor microenvironment biomarkers and clinical activity of ipilimumab in advanced melanoma
Source: J Transl Med. 2011 Nov 28;9:204. doi: 10.1186/1479-5876-9-204 (PMC3239318; doi:10.1186/1479-5876-9-204)
Supplement: Additional file 1 — Table S1. Specifications for antibodies used in IHC analyses. [file 1479-5876-9-204-S1.PDF]

Table S1 Specifications for antibodies used in IHC analyses.

Antibody	CD4	CD8	CD45RO	Granzyme B	IDO	Perforin	FoxP3
**Vendor**	Lab Vision	Lab Vision	Lab Vision	Abcam	Millipore Upstate	Millipore Chemicon	Abcam
**Catalog No.**	MS-492-S	MS-457-S	MS-112-P	ab4059	05-840	MAB4616	ab20034
**Lot No.**	392S707A	457S609E	112P611E	320013	32352	0604027513	422471
**Concentration**	Not given	Not given	0.2 mg/mL	Not given	0.463 mg/mL	0.2 mg/mL	1.0 mg/mL
**Date Received**	09/19/2007	09/19/2007	09/06/2007	09/06/2007	09/11/2007	09/11/2007	04/18/2008
**Form**	Monoclonal	Monoclonal	Monoclonal	Polyclonal	Monoclonal	Monoclonal	Monoclonal
**Source/isotype**	MsIgG1	MsIgG1(k)	MsIgG2a	RbIgG	MsIgG1	MsIgG2b	MsIgG1
**Clone**	1F6	C8/144B	UCHL-1	NA	10.1	δG9	236A/E7
**Target Antigen**	CD4 External Domain	CD8 C-terminus	IL-2 dependent T-cells	Polyclonal	IDO	YT lymphoma cell line	FoxP3
**Control Tissue**	Tonsil Q1777	Tonsil Q1777	Tonsil Q1777	Spleen Q1517	Sarcoidosis Q3755	Sarcoidosis Q3775	Tonsil Q1777
**Paraffin Reactive**	Yes	Yes	Yes	Yes	Yes	Yes	Yes
**Suggested Dilution**	1:150 (MsIgG1 @ 2.0 μg/ml)	1:200 (MsIgG1(k) @ 1.0 μg/ml)	1:3000 (MsIgG2a @ 0.5 μg/ml)	1:100 (RbIgG @ 1.0 μg/ml)	3.0 μg/ml	1.0 μg/ml	2.0 μg/ml

Incubation Time	Overnight	1-hour	1-hour	1-hour	1-hour	1-hour	Overnight
**Pretreatment**	SHIER1 + enzyme (1:40)	SHIER1 no enzyme	SHIER1 no enzyme	SHIER7 + enzyme (1:40)	SHIER8 + enzyme (1:40)	SHIER8 + enzyme (1:40)	SHIER2 no enzyme
**Detection System**	ABC	ABC	ABC	UV	UV	UV	ABC
**Subcellular Localization**	Membranous	Membranous	Membranous	Cytoplasmic	Cytoplasmic	Cytoplasmic	Nuclear

ABC = Avidin biotin-complex
