# Supplementary material for: A prospective phase II trial exploring the association between tumor microenvironment biomarkers and clinical activity of ipilimumab in advanced melanoma
Source: J Transl Med. 2011 Nov 28;9:204. doi: 10.1186/1479-5876-9-204 (PMC3239318; doi:10.1186/1479-5876-9-204)
Supplement: Additional file 2 — Table S2. Specifications Scoring of IHC and H&E-stained samples. [file 1479-5876-9-204-S2.PDF]

Table S2 Scoring of IHC and H&E-stained samples.

Biomarker	Scoring Method
**Tumor-infiltrating lymphocytes (TILs)**	3-level scale (in increments of 1) based upon percentage of the tumor containing TILs, as revealed by H&E staining: >50%, ≤50%, absent.
**CD45RO**	0-4 score (in increments of 0.5) based upon microscopic examination of the relative amount of $CD45RO^+$ cells in the sample
**CD4**	0-4 score (in increments of 0.5) based upon percentage of $CD4^+$ cells relative to the CD45Ro score
**CD8**	0-4 score (in increments of 0.5) based upon percentage of $CD8^+$ cells relative to the CD45Ro score
**Granzyme B**	0-4 score (in increments of 0.5) based upon microscopic examination of the relative amount of granzyme B-positive cells
**IDO**	0-4 score (in increments of 0.5) based upon microscopic examination of the relative amount of IDO-positive tumor cells
**Perforin**	0-4 score (in increments of 0.5) based upon microscopic examination of the relative amount of perforin-positive cells
**FoxP3**	0-4 score (in increments of 0.5) based upon microscopic examination of the relative amount of FoxP3-positive cells

0-4 scoring: 0 indicates very little or no staining and 4 indicates abundant number of positive-staining cells relative to the entire data set.
