# Supplementary material for: A prospective phase II trial exploring the association between tumor microenvironment biomarkers and clinical activity of ipilimumab in advanced melanoma
Source: J Transl Med. 2011 Nov 28;9:204. doi: 10.1186/1479-5876-9-204 (PMC3239318; doi:10.1186/1479-5876-9-204)
Supplement: Additional file 3 — Table S3. Joint frequencies of clinical activity and change from baseline of tumor biopsy H&E scores: TILs. [file 1479-5876-9-204-S3.PDF]

Table S3 Joint frequencies of clinical activity and change from baseline of tumor biopsy H&E scores: TILs.

Clinical Activity	Change from Baseline*					
	-2	-1	0	1	2	Total
**Benefit, n (%)**	0 (0.0)	0 (0.0)	3 (42.9)	3 (42.9)	1 (14.3)	7 (22.6)
**Non-benefit, n (%)**	0 (0.0)	3 (15.0)	15 (75.0)	2 (10.0)	0 (0.0)	20 (64.5)
**Unknown, n (%)**	0 (0.0)	0 (0.0)	3 (75.0)	1 (25.0)	0 (0.0)	4 (12.9)
**Total, n (%)**	0 (0.0)	3 (9.7)	21 (67.7)	6 (19.4)	1 (3.2)	31 (100)

Scores coded as 0 = Absent; 1 = Present ≤ 50%; 2 = Present > 50%.
*Change from baseline = posttreatment biopsy (1-5 weeks after first dose) score – pretreatment biopsy (screening) score.
H&E: hematoxylin and eosin; TILs: tumor-infiltrating lymphocytes.
