# Supplementary material for: A prospective phase II trial exploring the association between tumor microenvironment biomarkers and clinical activity of ipilimumab in advanced melanoma
Source: J Transl Med. 2011 Nov 28;9:204. doi: 10.1186/1479-5876-9-204 (PMC3239318; doi:10.1186/1479-5876-9-204)
Supplement: Additional file 4 — Table S4. Joint frequencies of clinical activity and pretreatment tumor biopsy IHC scores: FoxP3. [file 1479-5876-9-204-S4.PDF]

Table S4 Joint frequencies of clinical activity and pretreatment tumor biopsy IHC scores: FoxP3.

Clinical Activity	Pretreatment* Score							
	0.0	0.5	1.0	1.5	2.0	2.5	3.0	Total
Benefit, n (%)	2 (25.0)	3 (37.5)	2 (25.0)	0 (0.0)	0 (0.0)	1 (12.5)	0 (0.0)	8 (20.5)
Non-benefit, n (%)	16 (64.0)	7 (28.0)	2 (8.0)	0 (0.0)	0 (0.0)	0 (0.0)	0 (0.0)	25 (64.1)
Unknown, n (%)	2 (33.3)	1 (16.7)	3 (50.0)	0 (0.0)	0 (0.0)	0 (0.0)	0 (0.0)	6 (15.4)
Total, n (%)	20 (51.3)	11 (28.2)	7 (17.9)	0 (0.0)	0 (0.0)	1 (2.6)	0 (0.0)	39 (100)

*Pretreatment = screening biopsy (0-4 weeks before first dose, inclusive).
IHC: immunohistochemistry.
