# Supplementary material for: A prospective phase II trial exploring the association between tumor microenvironment biomarkers and clinical activity of ipilimumab in advanced melanoma
Source: J Transl Med. 2011 Nov 28;9:204. doi: 10.1186/1479-5876-9-204 (PMC3239318; doi:10.1186/1479-5876-9-204)
Supplement: Additional file 5 — Table S5. Joint frequencies of clinical activity and pretreatment tumor biopsy IHC scores: FoxP3. Joint frequencies of clinical activity and pretreatment tumor biopsy IHC scores: IDO. [file 1479-5876-9-204-S5.PDF]

Table S5 Joint frequencies of clinical activity and pretreatment tumor biopsy IHC scores: IDO.

Clinical Activity	Pretreatment* Score							
	**0.0**	**0.5**	**1.0**	**1.5**	**2.0**	**2.5**	**3.0**	**Total**
**Benefit, n (%)**	5 (62.5)	0 (0.0)	2 (25.0)	0 (0.0)	0 (0.0)	0 (0.0)	1 (12.5)	8 (19.5)
**Non-benefit, n (%)**	24 (88.9)	2 (7.4)	1 (3.7)	0 (0.0)	0 (0.0)	0 (0.0)	0 (0.0)	27 (65.9)
**Unknown, n (%)**	5 (83.3)	1 (16.7)	0 (0.0)	0 (0.0)	0 (0.0)	0 (0.0)	0 (0.0)	6 (14.6)
**Total, n (%)**	34 (82.9)	3 (7.3)	3 (7.3)	0 (0.0)	0 (0.0)	0 (0.0)	1 (2.4)	41 (100)

*Pretreatment = screening biopsy (0-4 weeks before first dose, inclusive).
IDO: indoleamine 2,3-dioxygenase; IHC: immunohistochemistry.
