# Supplementary material for: A prospective phase II trial exploring the association between tumor microenvironment biomarkers and clinical activity of ipilimumab in advanced melanoma
Source: J Transl Med. 2011 Nov 28;9:204. doi: 10.1186/1479-5876-9-204 (PMC3239318; doi:10.1186/1479-5876-9-204)
Supplement: Additional file 6 — Figure S1. mRNA Expression in tumor biopsies: scatter plot summarizing magnitude and significance of changes from baseline. [file 1479-5876-9-204-S6.PDF]

Figure S1 mRNA Expression in tumor biopsies: scatter plot summarizing magnitude and significance of changes from baseline.

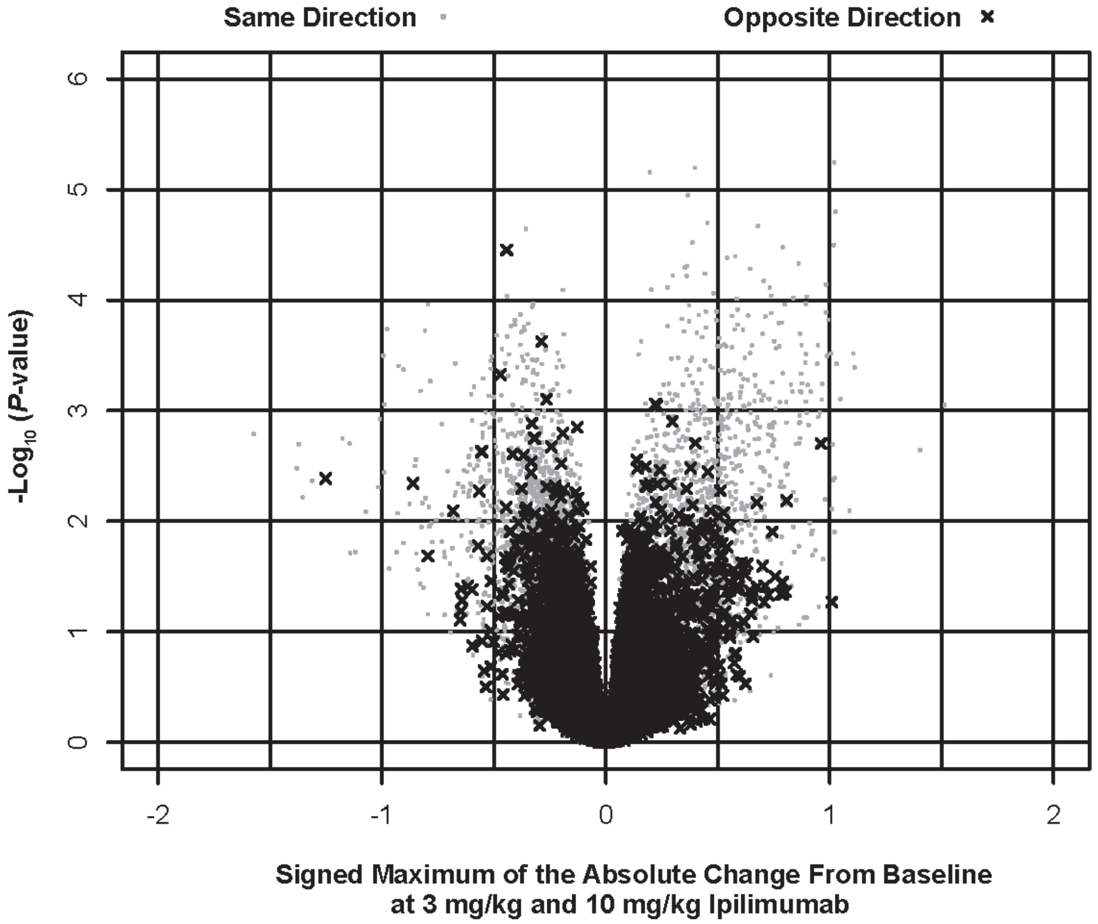

Pretreatment = 0 to 4 weeks before first dose, inclusive.
Posttreatment = 1 to 5 weeks since first dose, inclusive.
Same direction = changes from baseline at 3 mg/kg and 10 mg/kg ipilimumab are in the same direction.
Opposite direction = changes from baseline at 3 mg/kg and 10 mg/kg ipilimumab are in the opposite direction.
Each 1-unit change on the x-axis represents a 2-fold change from baseline in normalized expression level.
P-value for each probeset is from an F-test of the null hypothesis that there is no average change from baseline and no difference of such change between the 3 mg/kg and 10 mg/kg ipilimumab groups.
Each 1-unit increase on the y-axis represents a 10-fold decrease in *P*-value.
Each plotting symbol represents 1 probeset.
