# Supplementary material for: A prospective phase II trial exploring the association between tumor microenvironment biomarkers and clinical activity of ipilimumab in advanced melanoma
Source: J Transl Med. 2011 Nov 28;9:204. doi: 10.1186/1479-5876-9-204 (PMC3239318; doi:10.1186/1479-5876-9-204)
Supplement: Additional file 7 — Table S6. Model estimates for probe sets with time effect q-value < 0.05 and expression level increased from baseline. [file 1479-5876-9-204-S7.PDF]

Table S6 Model estimates for probe sets with time effect q-value <0.05 and expression level increased from baseline.

Probe Set ID	Gene Symbol	Gene Name	Time Effect 3 mg/kg	Time Effect 10 mg/kg	Dose Effect Pre-Tx	Dose Effect Post-Tx	Interaction Effect	Time Effect *P*-value	Dose Effect *P*-value	Interaction *P*-value	Time Effect q-value	Dose Effect q-value	Interaction q-value
202917_s_at	S100A8	S100 calcium binding protein A8	1.514	1.221	0.697	0.404	–0.293	0.001	0.344	0.672	0.035	0.523	0.857
217022_s_at	IGH@[I]	immunoglobulin heavy constant gamma 1	1.404	1.07	1.219	0.884	–0.334	0.002	0.025	0.623	0.049	0.453	0.851
203828_s_at	IL32[I]	interleukin 32	1.018	0.84	0.675	0.497	–0.178	<.001	0.061	0.633	0.025	0.453	0.853
203535_at	S100A9	S100 calcium binding protein A9	0.969	1.031	0.125	0.188	0.063	<.001	0.661	0.893	0.028	0.607	0.887
212543_at	AIM1[M]	absent in melanoma 1	0.966	0.359	0.492	–0.115	–0.607	0.001	0.137	0.092	0.032	0.46	0.796
203645_s_at	CD163	CD163 molecule	0.894	0.776	0.86	0.742	–0.118	<.001	0.045	0.761	0.028	0.453	0.868
34210_at	CD52	CD52 molecule	0.894	0.725	0.502	0.333	–0.169	<.001	0.121	0.631	0.025	0.458	0.853
212242_at	TUBA4A	tubulin, alpha 4a	0.892	0.46	0.338	–0.094	–0.431	<.001	0.226	0.2	0.028	0.489	0.797
205831_at	CD2[I]	CD2 molecule	0.891	0.948	0.483	0.54	0.057	<.001	0.279	0.892	0.027	0.504	0.886
211796_s_at	TRBC1[I]	T cell receptor beta constant 1	0.888	1.006	0.502	0.62	0.118	<.001	0.228	0.787	0.028	0.49	0.87
205081_at	CRIP1	cysteine-rich protein 1 (intestinal)	0.876	0.439	0.488	0.05	–0.438	0.001	0.107	0.198	0.032	0.458	0.797
213539_at	CD3D[I]	CD3d molecule, delta (CD3-TCR complex)	0.873	0.898	0.388	0.412	0.025	<.001	0.279	0.948	0.025	0.505	0.892
202238_s_at	NNMT	nicotinamide N-methyltransferase	0.869	0.173	0.933	0.237	–0.697	0.002	0.012	0.043	0.045	0.453	0.796
205569_at	LAMP3	lysosomal-associated membrane protein 3	0.868	0.124	0.108	–0.636	–0.743	0.001	0.062	0.025	0.039	0.453	0.796
213193_x_at	TRBC1[I]	T cell receptor beta constant 1	0.868	0.945	0.402	0.48	0.078	<.001	0.305	0.855	0.028	0.512	0.881
202688_at	TNFSF10[I]	tumor necrosis factor (ligand) superfamily, member 10	0.867	0.738	0.922	0.792	–0.129	<.001	0.022	0.72	0.025	0.453	0.865
202237_at	NNMT	nicotinamide N-methyltransferase	0.866	0.223	0.961	0.319	–0.643	0.001	0.008	0.053	0.038	0.453	0.796
210164_at	GZMB[I]	granzyme B	0.844	1.112	0.031	0.299	0.268	<.001	0.662	0.564	0.029	0.607	0.841
205242_at	CXCL13[I]	chemokine (C-X-C motif) ligand 13	0.821	0.864	0.302	0.345	0.043	0.001	0.615	0.921	0.039	0.595	0.888
206134_at	ADAMDEC1[I]	ADAM-like, decysin 1	0.808	0.864	0.289	0.345	0.056	0.002	0.39	0.901	0.046	0.536	0.887
221541_at	CRISPLD2	cysteine-rich secretory protein LCCL domain containing 2	0.807	0.195	0.432	–0.180	–0.613	0.001	0.1	0.05	0.039	0.457	0.796
215049_x_at	CD163	CD52 molecule	0.806	0.753	0.929	0.875	–0.054	0.001	0.032	0.89	0.035	0.453	0.886
205488_at	GZMA[I]	granzyme A	0.806	0.995	0.29	0.479	0.189	<.001	0.234	0.634	0.025	0.491	0.853
218805_at	GIMAP5[I]	GTPase, IMAP family member 5	0.799	0.632	0.459	0.292	–0.167	<.001	0.14	0.592	0.025	0.46	0.846
204661_at	CD52	CD52 molecule	0.785	0.756	0.413	0.384	–0.029	<.001	0.142	0.932	0.025	0.46	0.889

209083_at	CORO1A[I]	coronin, actin binding protein, 1A	0.767	0.984	0.178	0.396	0.218	<.001	0.306	0.555	0.025	0.512	0.84
204891_s_at	LCK[I]	lymphocyte-specific protein tyrosine kinase	0.764	0.776	0.231	0.243	0.012	<.001	0.475	0.973	0.027	0.557	0.892
204897_at	PTGER4[I]	prostaglandin E receptor 4 (subtype EP4)	0.754	0.44	0.544	0.231	–0.313	0.001	0.138	0.33	0.039	0.46	0.797
205159_at	CSF2RB	colony stimulating factor 2 receptor, beta, low-affinity (granulocyte-macrophage)	0.752	0.861	0.23	0.34	0.109	<.001	0.157	0.74	0.025	0.464	0.866
204438_at	MRC1	mannose receptor, C type 1	0.752	0.707	0.585	0.54	–0.045	0.002	0.241	0.908	0.045	0.495	0.888
209656_s_at	TMEM47	transmembrane protein 47	0.749	0.075	0.543	–0.131	–0.674	0.002	0.12	0.024	0.047	0.458	0.796
209671_x_at	TRA@	T cell receptor alpha	0.747	0.874	0.251	0.379	0.127	<.001	0.382	0.731	0.027	0.533	0.866
209355_s_at	PPAP2B	phosphatidic acid phosphatase type 2B	0.746	0.117	0.718	0.09	–0.628	0.002	0.053	0.034	0.046	0.453	0.796
201798_s_at	FER1L3	myoferlin	0.744	0.299	0.451	0.006	–0.445	0.001	0.373	0.139	0.041	0.53	0.796
206666_at	GZMK[I]	granzyme K	0.739	0.874	0.495	0.631	0.136	0.001	0.165	0.732	0.034	0.468	0.866
202687_s_at	TNFSF10[I]	tumor necrosis factor (ligand) superfamily, member 10	0.739	0.536	0.588	0.384	–0.203	<.001	0.057	0.472	0.025	0.453	0.829
210915_x_at	TRBC1[I]	T cell receptor beta constant 1	0.736	0.868	0.258	0.391	0.132	0.001	0.318	0.736	0.034	0.515	0.866
219243_at	GIMAP4[I]	GTPase, IMAP family member 4	0.733	0.72	0.609	0.596	–0.012	<.001	0.043	0.968	0.025	0.453	0.892
210140_at	CST7[I,C]	cystatin F (leukocystatin)	0.731	0.7	0.015	–0.015	–0.031	0.001	0.643	0.934	0.041	0.601	0.889
220532_s_at	TMEM176B	transmembrane protein 176B	0.727	0.58	1.014	0.867	–0.147	0.002	0.051	0.682	0.049	0.453	0.858
212226_s_at	PPAP2B	phosphatidic acid phosphatase type 2B	0.724	0.174	0.608	0.058	–0.550	0.002	0.077	0.054	0.044	0.454	0.796
211902_x_at	TRA@	T cell receptor alpha	0.722	0.774	0.178	0.23	0.052	<.001	0.464	0.876	0.025	0.554	0.884
204116_at	IL2RG[I]	interleukin 2 receptor, gamma	0.718	0.988	0.375	0.645	0.269	<.001	0.172	0.499	0.028	0.471	0.833
212646_at	RFTN1	raftlin, lipid raft linker 1	0.713	0.327	0.499	0.113	–0.386	<.001	0.056	0.119	0.025	0.453	0.796
204834_at	FGL2	fibrinogen-like 2	0.708	0.753	0.682	0.727	0.045	<.001	0.05	0.891	0.027	0.453	0.886
218345_at	TMEM176A	transmembrane protein 176A	0.706	0.452	1.001	0.747	–0.255	0.001	0.014	0.387	0.034	0.453	0.81
219506_at	C1orf54	chromosome 1 open reading frame 54	0.702	0.141	0.439	–0.122	–0.561	<.001	0.039	0.017	0.025	0.453	0.796
210321_at	GZMH[I]	granzyme H (cathepsin G-like 2, protein h-CCPX)	0.702	0.902	0.013	0.213	0.2	0.001	0.541	0.61	0.032	0.577	0.85
219777_at	GIMAP6[I]	GTPase, IMAP family member 6	0.697	0.482	0.623	0.409	–0.214	0.001	0.077	0.474	0.035	0.454	0.829
203761_at	SLA	Src-like-adaptor	0.696	0.827	0.348	0.479	0.131	0.001	0.106	0.722	0.032	0.458	0.865
204923_at	CXorf9[I]	SAM and SH3 domain containing 3	0.688	0.667	0.286	0.265	–0.021	0.001	0.279	0.952	0.041	0.504	0.892

208438_s_at	FGR	Gardner-Rasheed feline sarcoma viral (v-fgr) oncogene homolog	0.688	0.727	0.318	0.357	0.039	0.001	0.109	0.911	0.035	0.458	0.888
217388_s_at	KYNU	kynureninase (L-kynurenine hydrolase)	0.687	0.577	0.281	0.171	–0.110	0.001	0.489	0.724	0.034	0.561	0.866
205419_at	EBI2	G protein-coupled receptor 183	0.685	0.16	0.158	–0.368	–0.526	0.002	0.178	0.059	0.05	0.472	0.796
217838_s_at	EVL	Enah/Vasp-like	0.683	0.284	0.432	0.034	–0.399	0.002	0.271	0.158	0.045	0.504	0.796
221840_at	PTPRE	protein tyrosine phosphatase, receptor type, E	0.68	0.618	0.282	0.22	–0.062	<.001	0.336	0.806	0.025	0.52	0.872
210972_x_at	TRA@	T cell receptor alpha	0.678	0.834	0.24	0.396	0.156	0.001	0.386	0.674	0.034	0.535	0.857
1405_i_at	CCL5[I]	chemokine (C-C motif) ligand 5	0.675	0.849	0.326	0.5	0.174	0.002	0.343	0.672	0.046	0.522	0.857
204220_at	GMFG	glia maturation factor, gamma	0.674	0.706	0.342	0.374	0.032	<.001	0.22	0.913	0.025	0.487	0.888
214617_at	PRF1[I]	perforin 1 (pore forming protein)	0.673	1.106	–0.034	0.399	0.433	<.001	0.418	0.305	0.028	0.541	0.797
209670_at	TRAC	T cell receptor alpha constant	0.669	0.694	0.252	0.277	0.025	0.001	0.389	0.941	0.035	0.536	0.891
205291_at	IL2RB[I]	interleukin 2 receptor, beta	0.665	0.982	0.268	0.586	0.317	<.001	0.205	0.384	0.025	0.481	0.81
204890_s_at	LCK[I]	lymphocyte-specific protein tyrosine kinase	0.665	0.762	0.046	0.144	0.097	<.001	0.709	0.766	0.027	0.618	0.868
219385_at	SLAMF8	SLAM family member 8	0.664	0.739	0.34	0.415	0.075	<.001	0.198	0.801	0.025	0.479	0.871
204960_at	PTPRCAP	protein tyrosine phosphatase, receptor type, C-associated protein	0.66	0.839	0.037	0.215	0.178	<.001	0.487	0.579	0.025	0.561	0.843
220330_s_at	SAMSN1	SAM domain, SH3 domain and nuclear localization signals 1	0.652	0.665	0.51	0.523	0.013	0.001	0.092	0.969	0.035	0.454	0.892
207419_s_at	RAC2	ras-related C3 botulinum toxin substrate 2 (rho family, small GTP binding protein)	0.65	0.873	0.011	0.233	0.223	<.001	0.453	0.542	0.03	0.55	0.839
205495_s_at	GNLY[I]	granulysin	0.648	1.049	0.043	0.445	0.401	0.001	0.367	0.353	0.035	0.529	0.802
213915_at	NKG7[I]	natural killer cell group 7 sequence	0.646	0.956	0.029	0.339	0.31	0.001	0.544	0.453	0.038	0.578	0.826
204929_s_at	VAMP5	vesicle-associated membrane protein 5 (myobrevin)	0.64	0.437	0.335	0.132	–0.203	0.001	0.638	0.462	0.035	0.6	0.827
213620_s_at	ICAM2[I]	intercellular adhesion molecule 2	0.639	0.342	0.356	0.06	–0.296	0.002	0.088	0.288	0.047	0.454	0.797
217763_s_at	RAB31	RAB31, member RAS oncogene family	0.635	0.38	0.201	–0.054	–0.255	0.001	0.482	0.315	0.032	0.559	0.797
204655_at	CCL5	chemokine (C-C motif) ligand 5	0.628	0.739	0.364	0.475	0.111	0.002	0.281	0.759	0.044	0.506	0.868

207238_s_at	PTPRC	protein tyrosine phosphatase, receptor type, C	0.624	0.916	0.305	0.598	0.293	0.001	0.121	0.445	0.034	0.458	0.825
207651_at	GPR171	G protein-coupled receptor 171	0.622	0.792	0.046	0.217	0.171	<.001	0.596	0.548	0.025	0.591	0.84
202748_at	GBP2[I]	guanylate binding protein 2, interferon-inducible	0.621	0.544	0.516	0.439	–0.077	<.001	0.053	0.776	0.028	0.453	0.869
209276_s_at	GLRX	glutaredoxin (thioltransferase)	0.62	0.317	0.656	0.353	–0.303	0.001	0.025	0.226	0.036	0.453	0.797
206991_s_at	CCR5[I]	chemokine (C-C motif) receptor 5	0.615	0.61	0.374	0.369	–0.005	0.001	0.208	0.986	0.036	0.483	0.892
208296_x_at	TNFAIP8	tumor necrosis factor, alpha-induced protein 8	0.615	0.495	0.33	0.21	–0.120	0.001	0.282	0.656	0.032	0.506	0.856
212230_at	PPAP2B	phosphatidic acid phosphatase type 2B	0.613	0.154	0.638	0.179	–0.459	0.002	0.049	0.062	0.046	0.453	0.796
221087_s_at	APOL3	apolipoprotein L, 3	0.612	0.613	0.489	0.491	0.002	<.001	0.086	0.995	0.025	0.454	0.894
219386_s_at	SLAMF8	SLAM family member 8	0.61	0.732	0.405	0.527	0.121	0.001	0.168	0.728	0.04	0.469	0.866
205798_at	IL7R[I]	interleukin 7 receptor	0.606	0.585	0.037	0.017	–0.020	0.001	0.626	0.944	0.033	0.597	0.891
38149_at	ARHGAP25	Rho GTPase activating protein 25	0.602	0.457	0.363	0.218	–0.146	0.001	0.049	0.58	0.034	0.453	0.844
210260_s_at	TNFAIP8	tumor necrosis factor, alpha-induced protein 8	0.602	0.396	0.289	0.084	–0.205	<.001	0.27	0.4	0.03	0.503	0.813
205456_at	CD3E[I]	CD3e molecule, epsilon (CD3-TCR complex)	0.601	0.647	0.065	0.112	0.047	0.001	0.659	0.883	0.038	0.605	0.885
202524_s_at	SPOCK2	sparc/osteonectin, cwcv and kazal-like domains proteoglycan (testican) 2	0.6	0.521	0.2	0.121	–0.079	0.001	0.309	0.775	0.035	0.513	0.869
205992_s_at	IL15[I]	interleukin 15	0.596	0.28	0.198	–0.118	–0.316	0.002	0.576	0.219	0.049	0.587	0.797
208091_s_at	ECOP[C]	vesicular, overexpressed in cancer, prosurvival protein 1	0.59	0.442	0.375	0.228	–0.147	<.001	0.05	0.517	0.025	0.453	0.835
212588_at	PTPRC	protein tyrosine phosphatase, receptor type, C	0.59	0.759	0.293	0.462	0.169	0.001	0.118	0.621	0.037	0.458	0.851
213603_s_at	RAC2	ras-related C3 botulinum toxin substrate 2 (rho family, small GTP binding protein)	0.59	0.677	0.353	0.44	0.087	0.001	0.108	0.788	0.039	0.458	0.871
212587_s_at	PTPRC	protein tyrosine phosphatase, receptor type, C	0.589	0.778	0.35	0.54	0.189	0.001	0.122	0.581	0.035	0.458	0.844
204204_at	SLC31A2	solute carrier family 31 (copper transporters), member 2	0.589	0.364	0.468	0.243	–0.225	0.001	0.108	0.374	0.039	0.458	0.809
219528_s_at	BCL11B[I]	B-cell CLL/lymphoma	0.585	0.38	0.068	–0.137	–0.205	<.001	0.467	0.334	0.025	0.554	0.797

		11B (zinc finger protein)										
207979_s_at	CD8B[I]	CD8b molecule	0.583	0.522	0.05	–0.011	–0.061	0.001	0.849	0.827	0.038	0.653	0.875
210029_at	INDO[C]	indoleamine 2,3-dioxygenase 1	0.581	1.015	–0.012	0.421	0.433	0.002	0.576	0.328	0.046	0.587	0.797
212067_s_at	C1R[I]	complement component 1, r subcomponent	0.579	0.332	0.848	0.602	–0.247	0.002	0.068	0.329	0.045	0.454	0.797
203233_at	IL4R[I]	interleukin 4 receptor	0.579	0.351	0.198	–0.030	–0.228	<.001	0.091	0.245	0.025	0.454	0.797
201502_s_at	NFKBIA[I]	nuclear factor of kappa light polypeptide gene enhancer in B-cells inhibitor, alpha	0.564	0.43	0.302	0.168	–0.134	<.001	0.149	0.543	0.025	0.461	0.839
210031_at	CD247[I]	CD247 molecule (TCR component)	0.563	0.791	0.044	0.272	0.228	<.001	0.457	0.484	0.03	0.552	0.831
37145_at	GNLY[I]	granulysin	0.562	0.965	0.026	0.429	0.403	0.001	0.337	0.303	0.035	0.521	0.797
219812_at	PVRIG	poliovirus receptor related immunoglobulin domain containing	0.561	0.547	0.057	0.042	–0.015	0.001	0.693	0.958	0.035	0.613	0.892
218035_s_at	RBM47	RNA binding motif protein 47	0.561	0.603	0.489	0.532	0.043	0.001	0.02	0.887	0.039	0.453	0.886
204908_s_at	BCL3[C]	B-cell CLL/lymphoma 3	0.557	0.441	0.347	0.231	–0.116	0.001	0.009	0.64	0.034	0.453	0.853
203760_s_at	SLA	Src-like-adaptor	0.557	0.684	0.149	0.276	0.127	0.001	0.296	0.677	0.034	0.509	0.858
206715_at	TFEC	transcription factor EC	0.556	0.485	0.214	0.142	–0.071	0.002	0.438	0.797	0.045	0.547	0.871
204254_s_at	VDR	vitamin D (1,25-dihydroxyvitamin D3) receptor	0.556	0.232	0.204	–0.120	–0.324	0.001	0.311	0.131	0.035	0.513	0.796
202957_at	HCLS1	hematopoietic cell-specific Lyn substrate 1	0.55	0.591	0.512	0.553	0.041	0.002	0.056	0.894	0.048	0.453	0.887
204048_s_at	PHACTR2	phosphatase and actin regulator 2	0.55	0.061	0.252	–0.237	–0.489	<.001	0.017	0.012	0.029	0.453	0.796
217762_s_at	RAB31	RAB31, member RAS oncogene family	0.55	0.366	0.243	0.059	–0.184	0.001	0.52	0.43	0.035	0.571	0.822
205660_at	OASL[I]	2'-5'-oligoadenylate synthetase-like	0.547	0.397	0.085	–0.065	–0.150	0.001	0.828	0.549	0.041	0.647	0.84
203471_s_at	PLEK	pleckstrin	0.546	0.894	0.359	0.707	0.348	0.001	0.063	0.344	0.035	0.453	0.8
203508_at	TNFRSF1B[I]	tumor necrosis factor receptor superfamily, member 1B	0.546	0.529	0.24	0.223	–0.017	0.001	0.184	0.951	0.039	0.472	0.892
209685_s_at	PRKCB1	PRKCB protein kinase C, beta	0.545	0.668	0.126	0.249	0.123	<.001	0.286	0.671	0.03	0.507	0.857
204613_at	PLCG2[I]	phospholipase C, gamma 2 (phosphatidylinositol-specific)	0.543	0.462	0.329	0.248	–0.081	<.001	0.014	0.694	0.025	0.453	0.861
209732_at	CLEC2B[I]	C-type lectin domain family 2, member B	0.537	0.297	0.248	0.007	–0.241	0.001	0.525	0.292	0.041	0.572	0.797
210038_at	PRKCQ[I]	protein kinase C, theta	0.531	0.505	–0.137	–0.163	–0.026	<.001	0.82	0.915	0.03	0.646	0.888
214038_at	CCL8[I]	chemokine (C-C motif) ligand 8	0.529	0.948	0.246	0.665	0.419	0.001	0.18	0.285	0.038	0.472	0.797

211661_x_at	PTAFR[I]	platelet-activating factor receptor	0.529	0.653	0.072	0.196	0.125	<.001	0.46	0.644	0.027	0.552	0.853
210222_s_at	RTN1	reticulon 1	0.529	0.489	–0.390	–0.430	–0.040	<.001	0.611	0.863	0.027	0.594	0.882
219191_s_at	BIN2	bridging integrator 2	0.526	0.669	0.091	0.234	0.143	0.001	0.181	0.624	0.033	0.472	0.851
204224_s_at	GCH1[I]	GTP cyclohydrolase 1	0.524	1.026	0.478	0.981	0.502	<.001	0.017	0.109	0.025	0.453	0.796
210538_s_at	BIRC3	baculoviral IAP repeat-containing 3	0.519	0.399	0.249	0.129	–0.120	0.001	0.448	0.617	0.04	0.549	0.85
203528_at	SEMA4D	Semaphorin 4D	0.517	0.488	0.131	0.102	–0.029	0.001	0.243	0.91	0.038	0.495	0.888
221565_s_at	FAM26B	calcium homeostasis modulator 2	0.513	0.163	0.283	–0.067	–0.350	0.002	0.395	0.092	0.045	0.538	0.796
205403_at	IL1R2[I]	interleukin 1 receptor, type II	0.513	0.671	–0.125	0.033	0.158	0.001	0.906	0.608	0.039	0.665	0.85
206060_s_at	PTPN22[I]	protein tyrosine phosphatase, non-receptor type 22 (lymphoid)	0.512	0.375	0.168	0.032	–0.136	0.001	0.322	0.558	0.039	0.516	0.841
205404_at	HSD11B1	hydroxysteroid (11-beta) dehydrogenase 1	0.508	1.021	0.658	1.171	0.513	<.001	0.012	0.081	0.022	0.453	0.796
212647_at	RRAS	related RAS viral oncogene homolog	0.501	0.164	0.251	–0.086	–0.337	0.001	0.156	0.087	0.039	0.464	0.796
206804_at	CD3G[I]	CD3g molecule, gamma (CD3-TCR complex)	0.498	0.541	0.052	0.095	0.043	0.001	0.685	0.87	0.037	0.611	0.883
219259_at	SEMA4A	semaphorin 4A	0.498	0.476	–0.039	–0.061	–0.022	<.001	0.711	0.922	0.027	0.618	0.888
204858_s_at	ECGF1	thymidine phosphorylase	0.496	0.347	0.192	0.043	–0.149	0.001	0.612	0.488	0.035	0.594	0.831
64064_at	GIMAP5	GTPase, IMAP family member 5	0.494	0.447	0.29	0.243	–0.048	<.001	0.11	0.818	0.025	0.458	0.875
206687_s_at	PTPN6	protein tyrosine phosphatase, non-receptor type 6	0.494	0.614	0.266	0.386	0.121	0.002	0.137	0.69	0.049	0.46	0.86
208018_s_at	HCK	hemopoietic cell kinase	0.493	0.743	0.214	0.464	0.25	0.002	0.206	0.459	0.047	0.482	0.827
205692_s_at	CD38[I]	CD38 molecule	0.484	0.897	0.193	0.606	0.413	0.001	0.126	0.258	0.038	0.46	0.797
38964_r_at	WAS	Wiskott-Aldrich syndrome (eczema-thrombocytopenia)	0.483	0.511	0.205	0.233	0.028	<.001	0.111	0.903	0.027	0.458	0.887
214765_s_at	ASAHL	N-acylethanolamine acid amidase	0.482	0.422	0.293	0.234	–0.059	<.001	0.124	0.757	0.025	0.458	0.868
209879_at	SELPLG[I]	selectin P ligand	0.482	0.497	0.215	0.23	0.015	0.002	0.184	0.954	0.047	0.473	0.892
203920_at	NR1H3	nuclear receptor subfamily 1, group H, member 3	0.479	0.472	0.413	0.406	–0.008	<.001	0.116	0.973	0.029	0.458	0.892
216920_s_at	TARP	T cell receptor gamma variable 9; T cell receptor gamma constant 1	0.478	0.747	–0.023	0.246	0.268	0.001	0.534	0.407	0.039	0.574	0.815
201565_s_at	ID2[I]	inhibitor of DNA binding 2, dominant negative helix-loop-helix protein	0.467	0.265	0.271	0.069	–0.202	<.001	0.51	0.264	0.029	0.567	0.797

209606_at	PSCDBP	CYTIP cytohesin 1 interacting protein	0.461	0.666	0.034	0.239	0.205	0.001	0.22	0.487	0.039	0.487	0.831
207697_x_at	LILRB2[I]	leukocyte immunoglobulin-like receptor, subfamily B, member 2	0.46	0.556	0.102	0.198	0.096	0.001	0.34	0.711	0.038	0.521	0.864
213716_s_at	SECTM1[I]	secreted and transmembrane 1	0.453	0.55	0.173	0.27	0.098	0.001	0.249	0.694	0.034	0.497	0.861
211639_x_at	ARPC1B	actin related protein 2/3 complex, subunit 1B	0.447	0.638	0.29	0.48	0.191	0.001	0.088	0.506	0.042	0.454	0.834
210116_at	SH2D1A[I]	SH2 domain protein 1A	0.447	0.575	0.024	0.152	0.129	<.001	0.552	0.574	0.025	0.58	0.842
201288_at	ARHGDIB	Rho GDP dissociation inhibitor (GDI) beta	0.445	0.513	0.13	0.198	0.068	<.001	0.606	0.764	0.029	0.593	0.868
206637_at	P2RY14[I]	purinergic receptor P2Y, G-protein coupled, 14	0.444	0.353	0.182	0.09	–0.091	0.001	0.418	0.653	0.037	0.541	0.855
208436_s_at	IRF7[I]	interferon regulatory factor 7	0.443	0.347	0.231	0.135	–0.095	0.001	0.951	0.642	0.039	0.676	0.853
204852_s_at	PTPN7	protein tyrosine phosphatase, non-receptor type 7	0.443	0.42	0.099	0.076	–0.023	0.001	0.421	0.917	0.04	0.542	0.888
222062_at	IL27RA[I]	interleukin 27 receptor, alpha	0.442	0.384	0.109	0.051	–0.058	<.001	0.815	0.765	0.028	0.645	0.868
211469_s_at	CXCR6[I]	chemokine (C-X-C motif) receptor 6	0.437	0.588	0.016	0.166	0.151	<.001	0.635	0.51	0.025	0.599	0.835
206118_at	STAT4	signal transducer and activator of transcription 4	0.431	0.459	0.113	0.141	0.028	0.002	0.513	0.905	0.044	0.568	0.888
215806_x_at	TARP	T cell receptor gamma variable 9; T cell receptor gamma constant 1	0.431	0.666	–0.077	0.158	0.235	0.002	0.659	0.43	0.045	0.605	0.822
202531_at	IRF1[I,C]	interferon regulatory factor 1	0.429	0.732	0.203	0.505	0.303	0.001	0.211	0.308	0.035	0.483	0.797
218802_at	CCDC109B	coiled-coil domain containing 109B	0.424	0.318	0.013	–0.093	–0.106	0.001	0.882	0.578	0.038	0.659	0.843
220485_s_at	SIRPG[I]	signal-regulatory protein gamma (immunoglobulin)	0.421	0.636	0.066	0.282	0.216	<.001	0.372	0.359	0.025	0.53	0.803
215633_x_at	LST1[I]	leukocyte specific transcript 1	0.417	0.393	0.192	0.168	–0.023	0.002	0.278	0.914	0.045	0.504	0.888
202181_at	KIAA0247	---	0.416	0.38	0.204	0.168	–0.036	<.001	0.332	0.836	0.025	0.519	0.877
204153_s_at	MFNG	MFNG O-fucosylpeptide 3-beta-N-acetylglucosaminyltransferase	0.416	0.347	0.195	0.126	–0.069	0.001	0.311	0.718	0.035	0.513	0.865
206974_at	CXCR6[I]	chemokine (C-X-C motif) receptor 6	0.414	0.646	0.018	0.25	0.232	<.001	0.423	0.298	0.025	0.542	0.797
205349_at	GNA15	guanine nucleotide	0.412	0.282	0.051	–0.079	–0.130	0.001	0.754	0.476	0.039	0.629	0.83

		binding protein (G protein), alpha 15 (Gq class)											
218298_s_at	C14orf159	chromosome 14 open reading frame 159	0.405	0.137	0.229	–0.039	–0.268	0.001	0.164	0.076	0.034	0.468	0.796
209969_s_at	STAT1	signal transducer and activator of transcription 1, 91kDa	0.404	0.745	0.286	0.627	0.341	0.001	0.174	0.238	0.032	0.471	0.797
214084_x_at	LOC648998	---	0.403	0.581	0.175	0.352	0.178	0.002	0.085	0.512	0.049	0.454	0.835
211676_s_at	IFNGR1[I]	interferon gamma receptor 1	0.402	0.11	0.216	–0.076	–0.292	0.002	0.271	0.076	0.049	0.504	0.796
205936_s_at	HK3	hexokinase 3 (white cell)	0.401	0.438	0.054	0.092	0.037	0.001	0.338	0.859	0.036	0.521	0.882
211582_x_at	LST1[I]	leukocyte specific transcript 1	0.401	0.521	0.278	0.398	0.12	0.001	0.074	0.621	0.042	0.454	0.851
207681_at	CXCR3[I]	chemokine (C-X-C motif) receptor 3	0.4	0.412	0.086	0.098	0.012	0.001	0.71	0.953	0.034	0.618	0.892
31845_at	ELF4[I]	E74-like factor 4 (ets domain transcription factor)	0.398	0.393	0.111	0.105	–0.005	0.001	0.505	0.979	0.039	0.566	0.892
219690_at	TMEM149	transmembrane protein 149	0.398	0.446	0.023	0.071	0.048	<.001	0.878	0.798	0.025	0.658	0.871
211005_at	LAT[I]	linker for activation of T cells	0.397	0.402	–0.108	–0.103	0.005	0.001	0.868	0.982	0.038	0.657	0.892
209890_at	TSPAN5	tetraspanin 5	0.392	0.061	0.045	–0.287	–0.331	0.001	0.069	0.023	0.035	0.454	0.796
213295_at	CYLD	cylindromatosis (turban tumor syndrome)	0.391	0.151	0.127	–0.113	–0.240	0.002	0.246	0.139	0.046	0.496	0.796
206296_x_at	MAP4K1	mitogen-activated protein kinase kinase kinase kinase 1	0.391	0.43	–0.061	–0.022	0.04	0.002	0.919	0.859	0.048	0.668	0.882
206120_at	CD33	CD33 molecule	0.389	0.26	0.073	–0.056	–0.129	0.001	0.666	0.424	0.032	0.608	0.82
203143_s_at	KIAA0040		0.388	0.381	0.244	0.237	–0.007	0.001	0.026	0.973	0.037	0.453	0.892
208534_s_at	FLJ21767	RAS p21 protein activator 4 pseudogene	0.387	0.105	–0.049	–0.331	–0.282	<.001	0.039	0.016	0.025	0.453	0.796
214551_s_at	CD7[I]	CD7 molecule (immunoglobulin)	0.385	0.506	–0.022	0.099	0.122	<.001	0.749	0.547	0.026	0.628	0.839
203470_s_at	PLEK	pleckstrin	0.384	0.635	0.062	0.312	0.25	0.001	0.188	0.357	0.041	0.474	0.803
207375_s_at	IL15RA[I]	interleukin 15 receptor, alpha	0.38	0.488	0.231	0.34	0.108	0.001	0.178	0.617	0.035	0.472	0.85
214329_x_at	TNFSF10	tumor necrosis factor (ligand) superfamily, member 10	0.375	0.335	0.301	0.261	–0.040	0.001	0.114	0.826	0.039	0.458	0.875
211211_x_at	SH2D1A[I]	SH2 domain protein 1A	0.373	0.34	0.041	0.008	–0.033	<.001	0.768	0.833	0.025	0.634	0.877
221477_s_at	MGC5618	superoxide dismutase 2, mitochondrial	0.372	0.645	0.32	0.592	0.272	<.001	0.027	0.262	0.028	0.453	0.797
205965_at	BATF	basic leucine zipper transcription factor, ATF-like	0.368	0.572	0.1	0.304	0.204	<.001	0.393	0.369	0.029	0.537	0.807
220832_at	TLR8[I]	toll-like receptor 8	0.368	0.469	0.031	0.132	0.1	0.001	0.495	0.644	0.039	0.563	0.853

214054_at	DOK2	docking protein 2	0.367	0.286	0.125	0.045	–0.080	0.002	0.469	0.646	0.045	0.555	0.853
91703_at	EHBP1L1	EH domain binding protein 1-like 1	0.367	0.22	0.026	–0.121	–0.147	<.001	0.463	0.202	0.025	0.553	0.797
202727_s_at	IFNGR1	interferon gamma receptor 1	0.367	0.108	0.266	0.007	–0.260	0.001	0.242	0.074	0.042	0.495	0.796
211434_s_at	CCRL2[I]	chemokine (C-C motif) receptor-like 2	0.365	0.501	0.116	0.252	0.136	0.001	0.221	0.531	0.035	0.487	0.837
211339_s_at	ITK[I]	IL2-inducible T-cell kinase	0.365	0.465	–0.021	0.078	0.099	0.001	0.851	0.642	0.039	0.654	0.853
214049_x_at	CD7[I]	CD7 molecule (immunoglobulin)	0.363	0.487	0.004	0.128	0.124	0.002	0.771	0.586	0.045	0.634	0.846
221755_at	EHBP1L1	EH domain binding protein 1-like 1	0.361	0.299	–0.092	–0.154	–0.062	<.001	0.588	0.652	0.025	0.589	0.855
213137_s_at	PTPN2	protein tyrosine phosphatase, non-receptor type 2	0.359	0.102	0.222	–0.035	–0.257	0.001	0.018	0.06	0.037	0.453	0.796
210784_x_at	LILRB3[I]	leukocyte immunoglobulin-like receptor, subfamily B, member 3	0.358	0.563	0.146	0.351	0.205	<.001	0.062	0.341	0.027	0.453	0.8
35150_at	CD40[I]	CD40 molecule, TNF receptor superfamily member 5	0.35	0.411	0.028	0.089	0.061	0.001	0.783	0.748	0.035	0.637	0.867
207104_x_at	LILRB1[I]	leukocyte immunoglobulin-like receptor, subfamily B (with TM and ITIM domains), member 1	0.349	0.51	0.168	0.329	0.161	0.002	0.151	0.485	0.044	0.462	0.831
210354_at	IFNG[I,C]	interferon, gamma	0.347	0.546	0.028	0.228	0.2	0.001	0.527	0.383	0.035	0.573	0.81
211210_x_at	SH2D1A[I]	SH2 domain protein 1A	0.345	0.429	0.053	0.136	0.083	0.001	0.615	0.657	0.032	0.595	0.856
210659_at	CMKLR1[I]	chemokine-like receptor 1	0.344	0.152	0.118	–0.074	–0.192	0.002	0.299	0.179	0.045	0.51	0.797
201369_s_at	ZFP36L2	zinc finger protein 36, C3H type-like 2	0.342	0.132	0.067	–0.143	–0.209	<.001	0.182	0.072	0.025	0.472	0.796
212501_at	CEBPB[I]	CCAAT/enhancer binding protein (C/EBP), beta	0.333	0.251	0.26	0.178	–0.082	0.001	0.159	0.581	0.036	0.465	0.844
213733_at	MYO1F	myosin IF	0.333	0.54	–0.019	0.188	0.207	0.002	0.253	0.392	0.048	0.498	0.81
206765_at	KCNJ2	potassium inwardly-rectifying channel, subfamily J, member 2	0.33	0.378	0.162	0.21	0.048	0.001	0.768	0.788	0.037	0.634	0.871
220658_s_at	ARNTL2	aryl hydrocarbon receptor nuclear translocator-like 2	0.327	0.239	0.036	–0.052	–0.088	0.002	0.88	0.567	0.045	0.659	0.841
211862_x_at	CFLAR	CASP8 and FADD-like apoptosis regulator	0.325	0.373	0.101	0.15	0.048	<.001	0.372	0.772	0.03	0.53	0.869
201368_at	ZFP36L2	zinc finger protein 36, C3H type-like 2	0.325	0.081	0.185	–0.058	–0.243	0.001	0.087	0.056	0.042	0.454	0.796

209546_s_at	APOL1[I]	apolipoprotein L, 1	0.324	0.526	0.31	0.512	0.203	0.001	0.14	0.365	0.039	0.46	0.805
210663_s_at	KYNU[I]	kynureninase (L-kynurenine hydrolase)	0.321	0.349	–0.100	–0.071	0.028	<.001	0.969	0.849	0.025	0.68	0.88
207643_s_at	TNFRSF1A[I]	tumor necrosis factor receptor superfamily, member 1A	0.321	0.155	0.203	0.038	–0.166	0.002	0.2	0.229	0.047	0.48	0.797
210225_x_at	LILRB3[I]	leukocyte immunoglobulin-like receptor, subfamily B, member 3	0.32	0.453	0.137	0.269	0.132	0.002	0.129	0.524	0.045	0.46	0.835
203904_x_at	CD82[C]	CD82 molecule	0.319	0.034	0.116	–0.170	–0.285	0.001	0.092	0.014	0.034	0.454	0.796
219938_s_at	PSTPIP2	proline-serine-threonine phosphatase interacting protein 2	0.316	0.431	0.136	0.251	0.115	<.001	0.342	0.498	0.026	0.522	0.833
204789_at	FMNL1	formin-like 1	0.314	0.499	0.012	0.197	0.185	0.002	0.361	0.41	0.047	0.527	0.817
215706_x_at	ZYX	zyxin	0.312	0.305	–0.125	–0.132	–0.007	0.002	0.929	0.964	0.044	0.67	0.892
214219_x_at	MAP4K1	mitogen-activated protein kinase kinase kinase kinase 1	0.311	0.308	0.038	0.035	–0.003	0.001	0.711	0.983	0.034	0.618	0.892
204396_s_at	GRK5	G protein-coupled receptor kinase 5	0.304	0.107	0.01	–0.187	–0.197	0.001	0.215	0.1	0.038	0.486	0.796
218205_s_at	MKNK2	MAP kinase interacting serine/threonine kinase 2	0.299	0.142	0.198	0.041	–0.157	<.001	0.104	0.113	0.025	0.458	0.796
204804_at	TRIM21	tripartite motif-containing 21	0.299	0.339	–0.024	0.015	0.04	0.002	0.824	0.818	0.048	0.647	0.875
213475_s_at	ITGAL[I]	integrin, alpha L (antigen CD11A (p180), lymphocyte function-associated antigen 1; alpha polypeptide)	0.298	0.436	0.07	0.207	0.137	0.001	0.286	0.466	0.038	0.507	0.828
211868_x_at	IGHA1[I]	immunoglobulin heavy constant alpha 1	0.295	0.402	0.205	0.311	0.107	0.002	0.111	0.571	0.046	0.458	0.842
203620_s_at	FCHSD2	FCH and double SH3 domains 2	0.294	0.22	0.032	–0.042	–0.075	0.001	0.843	0.578	0.039	0.651	0.843
205863_at	S100A12	S100 calcium binding protein A12	0.292	0.329	–0.175	–0.139	0.037	0.002	0.995	0.824	0.045	0.685	0.875
221656_s_at	ARHGEF10L	Rho guanine nucleotide exchange factor (GEF) 10-like	0.286	0.069	0.204	–0.013	–0.218	0.001	0.1	0.047	0.038	0.457	0.796
213940_s_at	FNBP1	formin binding protein 1	0.283	0.254	–0.064	–0.093	–0.028	0.002	0.851	0.847	0.049	0.654	0.88
203490_at	ELF4	E74-like factor 4 (ets domain transcription factor)	0.282	0.237	0.091	0.046	–0.045	0.001	0.268	0.721	0.032	0.503	0.865
215223_s_at	SOD2	superoxide dismutase 2, mitochondrial	0.281	0.907	0.252	0.877	0.625	0.001	0.028	0.074	0.04	0.453	0.796
211316_x_at	CFLAR	CASP8 and FADD-like apoptosis regulator	0.28	0.444	–0.020	0.143	0.164	<.001	0.357	0.286	0.025	0.526	0.797

206181_at	SLAMF1[I]	signaling lymphocytic activation molecule family member 1	0.278	0.418	0.365	0.504	0.14	0.001	0.123	0.441	0.039	0.458	0.824
210817_s_at	CALCOCO2[I]	calcium binding and coiled-coil domain 2	0.276	0.151	0.238	0.114	–0.124	<.001	0.073	0.214	0.025	0.454	0.797
203110_at	PTK2B	PTK2B protein tyrosine kinase 2 beta	0.27	0.298	0.017	0.045	0.028	0.001	0.381	0.843	0.035	0.533	0.879
220358_at	BATF3	basic leucine zipper transcription factor, ATF-like 3	0.268	0.246	–0.033	–0.056	–0.023	0.002	0.896	0.867	0.044	0.663	0.883
218871_x_at	GALNACT-2	novel protein similar to chondroitin sulfate GalNAcT-2	0.263	0.023	0.165	–0.075	–0.240	0.002	0.061	0.021	0.047	0.453	0.796
210563_x_at	CFLAR	CASP8 and FADD-like apoptosis regulator	0.258	0.495	0.037	0.274	0.237	<.001	0.239	0.168	0.025	0.494	0.796
220933_s_at	ZCCHC6	zinc finger, CCHC domain containing 6	0.255	0.387	0.14	0.272	0.132	0.002	0.178	0.458	0.049	0.472	0.827
204961_s_at	NCF1	neutrophil cytosolic factor 1; neutrophil cytosolic factor 1C pseudogene	0.251	0.526	0.005	0.28	0.275	0.001	0.135	0.178	0.035	0.46	0.797
35254_at	TRAFD1	TRAF-type zinc finger domain containing 1	0.248	0.303	–0.061	–0.006	0.055	0.002	0.872	0.714	0.049	0.658	0.865
209880_s_at	SELPLG[I]	selectin P ligand	0.244	0.378	0.064	0.198	0.134	<.001	0.103	0.365	0.028	0.458	0.805
207351_s_at	SH2D2A	SH2 domain protein 2A	0.244	0.447	–0.055	0.148	0.203	<.001	0.495	0.195	0.025	0.563	0.797
207181_s_at	CASP7	caspase 7, apoptosis-related cysteine peptidase	0.242	0.237	0.079	0.074	–0.005	0.001	0.875	0.964	0.034	0.658	0.892
219541_at	LIME1[I]	Lck interacting transmembrane adaptor 1	0.241	0.454	0.151	0.365	0.214	<.001	0.059	0.131	0.025	0.453	0.796
209508_x_at	CFLAR	CASP8 and FADD-like apoptosis regulator	0.233	0.364	0.08	0.211	0.131	<.001	0.141	0.295	0.025	0.46	0.797
212663_at	FKBP15	FK506 binding protein 15, 133kDa	0.231	0.258	0.088	0.115	0.027	0.001	0.628	0.831	0.038	0.598	0.876
204152_s_at	MFNG	MFNG O-fucosylpeptide 3-beta-N-acetylglucosaminyltransferase	0.23	0.343	0.058	0.171	0.113	0.001	0.073	0.424	0.032	0.454	0.82
213857_s_at	CD47[I]	CD47 molecule	0.227	0.199	0.168	0.14	–0.028	0.002	0.294	0.806	0.046	0.509	0.872
208485_x_at	CFLAR	CASP8 and FADD-like apoptosis regulator	0.226	0.408	0.046	0.228	0.181	<.001	0.215	0.219	0.025	0.486	0.797
205205_at	RELB[I]	v-rel reticuloendotheliosis viral oncogene homolog B	0.226	0.355	<.001	0.129	0.129	<.001	0.579	0.29	0.025	0.587	0.797
210564_x_at	CFLAR	CASP8 and FADD-like apoptosis regulator	0.223	0.386	–0.020	0.143	0.163	<.001	0.367	0.264	0.028	0.529	0.797

217119_s_at	CXCR3[I]	chemokine (C-X-C motif) receptor 3	0.223	0.284	–0.028	0.033	0.061	0.001	0.681	0.625	0.034	0.611	0.851
211893_x_at	CD6[I]	CD6 molecule	0.22	0.295	–0.026	0.049	0.075	<.001	0.86	0.536	0.028	0.656	0.837
209039_x_at	EHD1	EH-domain containing 1	0.218	0.355	–0.163	–0.026	0.137	0.002	0.592	0.383	0.045	0.591	0.81
214486_x_at	CFLAR	CASP8 and FADD-like apoptosis regulator	0.216	0.277	0.09	0.15	0.06	<.001	0.216	0.563	0.025	0.486	0.841
205484_at	SIT1[I]	signaling threshold regulating transmembrane adaptor 1 (immunoglobulin)	0.215	0.276	0.08	0.141	0.061	0.002	0.189	0.639	0.044	0.474	0.853
210439_at	ICOS[I]	inducible T-cell co-stimulator	0.209	0.417	–0.011	0.197	0.208	<.001	0.275	0.184	0.029	0.504	0.797
214370_at	S100A8	S100 calcium binding protein A8	0.206	0.447	–0.053	0.188	0.241	0.002	0.232	0.205	0.049	0.491	0.797
48531_at	TNIP2	TNFAIP3 interacting protein 2	0.205	0.14	0.152	0.087	–0.065	<.001	0.259	0.384	0.025	0.5	0.81
222221_x_at	EHD1	EH-domain containing 1	0.201	0.372	–0.211	–0.039	0.171	<.001	0.25	0.184	0.025	0.497	0.797
202523_s_at	SPOCK2	sparc/osteonectin, cwcv and kazal-like domains proteoglycan (testican) 2	0.2	0.21	0.037	0.046	0.009	0.001	0.823	0.929	0.039	0.647	0.889
208373_s_at	P2RY6	pyrimidinergic receptor P2Y, G-protein coupled, 6	0.198	0.398	–0.004	0.197	0.2	<.001	0.206	0.083	0.022	0.482	0.796
216835_s_at	DOK1	docking protein 1	0.178	0.22	0.073	0.115	0.042	0.001	0.254	0.688	0.042	0.498	0.859
202837_at	TRAFD1	TRAF-type zinc finger domain containing 1	0.178	0.122	0.019	–0.037	–0.056	0.001	0.873	0.464	0.035	0.658	0.827
209939_x_at	CFLAR	CASP8 and FADD-like apoptosis regulator	0.175	0.361	–0.009	0.177	0.186	<.001	0.244	0.166	0.029	0.495	0.796
31826_at	FKBP15	FK506 binding protein 15, 133kDa	0.171	0.176	0.063	0.069	0.006	0.002	0.717	0.952	0.05	0.619	0.892
204007_at	FCGR3B[I]	Fc fragment of IgG, low affinity IIIb, receptor (CD16b)	0.165	0.446	0.008	0.289	0.281	0.002	0.067	0.116	0.045	0.453	0.796
220966_x_at	ARPC5L	actin related protein 2/3 complex, subunit 5-like	0.159	0.185	–0.041	–0.015	0.025	0.002	0.992	0.781	0.045	0.685	0.869
37170_at	BMP2K	BMP2 inducible kinase	0.159	0.03	0.023	–0.106	–0.129	<.001	0.065	0.017	0.027	0.453	0.796
204794_at	DUSP2	dual specificity phosphatase 2	0.159	0.229	–0.049	0.021	0.07	0.002	0.803	0.503	0.044	0.642	0.833
207794_at	CCR2[I,C]	chemokine (C-C motif) receptor 2	0.154	0.26	–0.107	–0.001	0.105	0.001	0.734	0.308	0.032	0.624	0.797
50400_at	PAOX	polyamine oxidase (exo-N4-amino)	0.147	0.123	0.121	0.097	–0.024	<.001	0.075	0.707	0.028	0.454	0.863
219620_x_at	C9orf167	chromosome 9 open reading frame 167	0.131	0.271	–0.013	0.127	0.14	0.002	0.503	0.224	0.048	0.565	0.797
202460_s_at	LPIN2	lipin 2	0.127	0.273	0.182	0.328	0.146	0.001	0.082	0.167	0.035	0.454	0.796
204949_at	ICAM3[I]	intercellular adhesion molecule 3	0.126	0.408	0.042	0.324	0.282	0.001	0.012	0.065	0.037	0.453	0.796
219971_at	IL21R[I]	interleukin 21 receptor	0.126	0.212	0.028	0.114	0.086	0.002	0.197	0.362	0.049	0.479	0.803

210448_s_at	P2RX5	purinergic receptor P2X, ligand-gated ion channel, 5	0.124	0.538	–0.020	0.393	0.413	<.001	0.012	0.022	0.027	0.453	0.796
213669_at	FCHO1	FCH domain only 1	0.118	0.203	–0.054	0.03	0.084	0.002	0.894	0.337	0.045	0.662	0.797
217001_x_at	HLA-DOA[I]	major histocompatibility complex, class II, DO alpha	0.118	0.125	0.082	0.089	0.007	0.002	0.135	0.918	0.048	0.46	0.888
209636_at	NFKB2[I]	nuclear factor of kappa light polypeptide gene enhancer in B-cells 2 (p49/p100)	0.118	0.145	–0.013	0.014	0.027	0.002	0.696	0.706	0.046	0.614	0.863
202887_s_at	DDIT4	DNA-damage-inducible transcript 4	0.106	0.509	–0.578	–0.175	0.403	0.001	0.005	0.03	0.035	0.453	0.796
217326_x_at	IL23A[I]	interleukin 23, alpha subunit p19	0.094	0.197	–0.004	0.098	0.103	<.001	0.181	0.072	0.022	0.472	0.796
205036_at	LSM6	LSM6 homolog, U6 small nuclear RNA associated	0.085	0.185	–0.106	–0.006	0.101	0.001	0.113	0.17	0.038	0.458	0.796
205922_at	VNN2	vanin 2	0.081	0.516	–0.070	0.365	0.435	0.001	0.02	0.024	0.039	0.453	0.796
205467_at	CASP10[I]	caspase 10, apoptosis-related cysteine peptidase	0.053	0.21	0.032	0.189	0.157	0.001	0.046	0.045	0.037	0.453	0.796
214681_at	GK	glycerol kinase 3 pseudogene; glycerol kinase	0.032	0.318	–0.064	0.222	0.286	0.001	0.032	0.012	0.034	0.453	0.796
217691_x_at	SLC16A3	solute carrier family 16, member 3 (monocarboxylic acid transporter 4)	0.013	0.262	–0.102	0.147	0.249	0.002	0.095	0.013	0.044	0.455	0.796
217167_x_at	GK	glycerol kinase 3 pseudogene; glycerol kinase	0.006	0.322	–0.148	0.169	0.317	<.001	0.014	0.004	0.028	0.453	0.796

I = immune related; C = cancer related; M = melanoma related
