# Supplementary material for: A prospective phase II trial exploring the association between tumor microenvironment biomarkers and clinical activity of ipilimumab in advanced melanoma
Source: J Transl Med. 2011 Nov 28;9:204. doi: 10.1186/1479-5876-9-204 (PMC3239318; doi:10.1186/1479-5876-9-204)
Supplement: Additional file 8 — Table S7. Model estimates for probe sets with time effect q-value < 0.05 and expression level decreased from baseline. [file 1479-5876-9-204-S8.PDF]

Table S7 Model estimates for probe sets with time effect q-value <0.05 and expression level decreased from baseline.

Probe Set ID	Gene Symbol	Gene Name	Time Effect 3 mg/kg	Time Effect 10 mg/kg	Dose Effect Pre-Tx	Dose Effect Post-Tx	Interaction Effect	Time Effect *P*-value	Dose Effect *P*-value	Interaction *P*-value	Time Effect q-value	Dose Effect q-value	Interaction q-value
205338_s_at	DCT[M]	tyrosinase-related protein-2	–1.572	–0.365	–0.878	0.328	1.207	0.002	0.148	0.052	0.044	0.461	0.796
206696_at	GPR143	G protein-coupled receptor 143	–1.372	–0.267	–1.055	0.051	1.105	0.002	0.076	0.044	0.046	0.454	0.796
220245_at	SLC45A2[M]	solute carrier family 45, member 2	–1.176	–0.393	–0.056	0.726	0.783	0.002	0.245	0.101	0.045	0.496	0.796
219464_at	CA14	carbonic anhydrase XIV	–1.144	–0.004	–1.000	0.14	1.14	0.002	0.043	0.012	0.046	0.453	0.796
219107_at	BCAN	brevican	–1.009	–0.132	–0.853	0.024	0.877	0.001	0.033	0.022	0.039	0.453	0.796
214475_x_at	CAPN3	calpain 3, (p94)	–0.995	–0.476	–0.705	–0.187	0.519	<.001	0.31	0.159	0.028	0.513	0.796
221644_s_at	SLC45A2	solute carrier family 45, member 2	–0.988	–0.237	–0.070	0.681	0.751	0.001	0.102	0.043	0.035	0.458	0.796
220425_x_at	ROPN1B	ropporin, rhophilin associated protein 1B	–0.976	–0.460	–0.222	0.294	0.516	<.001	0.363	0.136	0.025	0.527	0.796
211890_x_at	CAPN3	calpain 3, (p94)	–0.926	–0.389	–0.669	–0.132	0.537	<.001	0.236	0.116	0.029	0.492	0.796
204187_at	GMPR	guanosine monophosphate reductase	–0.902	–0.159	–0.483	0.26	0.743	<.001	0.023	0.02	0.029	0.453	0.796
210944_s_at	CAPN3	calpain 3, (p94)	–0.827	–0.338	–0.627	–0.138	0.489	0.001	0.274	0.122	0.034	0.504	0.796
91920_at	BCAN	brevican	–0.807	–0.262	–0.574	–0.029	0.545	<.001	0.057	0.048	0.025	0.453	0.796
210963_s_at	GYG2	glycogenin 2	–0.793	–0.040	–0.591	0.163	0.754	<.001	0.003	0.003	0.025	0.453	0.796
210964_s_at	GYG2	glycogenin 2	–0.783	–0.099	–0.598	0.087	0.685	0.001	0.013	0.015	0.032	0.453	0.796
205376_at	INPP4B	inositol polyphosphate-4-phosphatase, type II, 105kDa	–0.732	–0.099	–0.549	0.085	0.633	0.001	0.023	0.024	0.04	0.453	0.796
212445_s_at	NEDD4L	neural precursor cell expressed, developmentally down-regulated 4-like	–0.671	–0.279	–0.550	–0.158	0.393	<.001	0.137	0.111	0.028	0.46	0.796
209681_at	SLC19A2	solute carrier family 19 (thiamine transporter), member 2	–0.601	–0.252	–0.191	0.159	0.349	0.002	0.293	0.169	0.048	0.508	0.796
212807_s_at	SORT1	sortilin 1	–0.600	–0.379	–0.178	0.043	0.221	0.001	0.32	0.375	0.034	0.516	0.809
222258_s_at	SH3BP4	SH3-domain binding protein 4	–0.596	–0.373	–0.056	0.168	0.223	0.001	0.56	0.386	0.039	0.582	0.81
218402_s_at	HPS4	Hermansky-Pudlak syndrome 4	–0.551	–0.137	–0.399	0.014	0.414	0.001	0.064	0.054	0.04	0.453	0.796
203702_s_at	TTLL4	tubulin tyrosine ligase-like family, member 4	–0.550	–0.083	–0.360	0.107	0.467	0.002	0.071	0.029	0.044	0.454	0.796
222116_s_at	TBC1D16	TBC1 domain family, member 16	–0.536	–0.303	–0.277	–0.044	0.233	0.001	0.322	0.297	0.038	0.516	0.797
212632_at	STX7	syntaxin 7	–0.521	–0.317	–0.280	–0.076	0.204	0.001	0.144	0.343	0.034	0.46	0.8
208029_s_at	LAPTM4B	lysosomal protein	–0.519	–0.423	–0.264	–0.169	0.095	0.001	0.076	0.7	0.041	0.454	0.862

		transmembrane 4 beta										
209108_at	TSPAN6	tetraspanin 6	–0.514	–0.588	0.296	0.222	–0.074	0.001	0.815	0.781	0.033	0.645	0.869
220272_at	BNC2	basonuclin 2	–0.513	–0.234	–0.474	–0.195	0.28	<.001	0.048	0.141	0.028	0.453	0.796
205677_s_at	DLEU1	deleted in lymphocytic leukemia 1 (non-protein coding)	–0.505	–0.232	–0.043	0.23	0.273	0.001	0.368	0.178	0.038	0.529	0.797
216048_s_at	RHOBTB3	Rho-related BTB domain containing 3	–0.499	–0.461	–0.178	–0.139	0.039	0.001	0.358	0.87	0.035	0.526	0.883
202975_s_at	RHOBTB3	Rho-related BTB domain containing 3	–0.499	–0.508	–0.153	–0.162	–0.009	<.001	0.475	0.969	0.028	0.557	0.892
209238_at	STX3	syntaxin 3	–0.498	–0.355	–0.316	–0.174	0.143	0.001	0.367	0.521	0.038	0.529	0.835
201535_at	UBL3	ubiquitin-like 3	–0.494	–0.270	–0.195	0.029	0.224	0.002	0.285	0.296	0.045	0.507	0.797
202976_s_at	RHOBTB3	Rho-related BTB domain containing 3	–0.487	–0.398	–0.047	0.042	0.089	<.001	0.548	0.657	0.026	0.579	0.857
202946_s_at	BTBD3	BTB (POZ) domain containing 3	–0.484	–0.230	–0.344	–0.091	0.254	0.002	0.082	0.227	0.05	0.454	0.797
204040_at	RNF144A	ring finger protein 144A	–0.475	–0.244	–0.211	0.019	0.231	<.001	0.178	0.205	0.029	0.472	0.797
219641_at	DET1	de-etiolated homolog 1	–0.473	–0.280	–0.322	–0.129	0.193	0.001	0.116	0.338	0.039	0.458	0.798
211844_s_at	NRP2	neuropilin 2	–0.469	–0.223	–0.215	0.031	0.246	0.001	0.206	0.198	0.039	0.482	0.797
204527_at	MYO5A	myosin VA (heavy chain 12, myoxin)	–0.463	–0.489	0.004	–0.022	–0.026	0.002	0.67	0.919	0.047	0.609	0.888
203612_at	BYSL	bystin-like	–0.457	–0.134	–0.435	–0.112	0.323	0.002	0.019	0.074	0.042	0.453	0.796
212290_at	SLC7A1	solute carrier family 7 (cationic amino acid transporter, y+ system), member 1	–0.448	–0.126	–0.149	0.173	0.322	<.001	0.157	0.043	0.028	0.464	0.796
205673_s_at	ASB9	ankyrin repeat and SOCS box-containing 9	–0.441	–0.013	–0.453	–0.025	0.428	<.001	0.004	0.004	0.026	0.453	0.796
216059_at	PAX3	paired box 3	–0.437	–0.137	–0.343	–0.042	0.301	0.002	0.056	0.088	0.045	0.453	0.796
219051_x_at	METRN	meteorin, glial cell differentiation regulator	–0.436	–0.083	–0.472	–0.118	0.353	<.001	0.015	0.023	0.03	0.453	0.796
209109_s_at	TSPAN6	tetraspanin 6	–0.433	–0.409	0.238	0.262	0.024	<.001	0.725	0.903	0.028	0.621	0.887
204107_at	NFYA	nuclear transcription factor Y, alpha	–0.423	–0.031	–0.400	–0.009	0.392	<.001	0.001	0.005	0.025	0.453	0.796
202873_at	ATP6V1C1	ATPase, H+ transporting, lysosomal 42kDa, V1 subunit C1	–0.421	–0.247	–0.137	0.038	0.175	0.002	0.503	0.343	0.044	0.565	0.8
204109_s_at	NFYA	nuclear transcription factor Y, alpha	–0.417	–0.072	–0.433	–0.088	0.345	0.002	0.004	0.034	0.044	0.453	0.796
204992_s_at	PFN2	profilin 2	–0.407	–0.547	–0.240	–0.380	–0.139	0.001	0.199	0.546	0.032	0.48	0.839
202609_at	EPS8	epidermal growth factor receptor pathway substrate 8	–0.405	–0.348	0.035	0.092	0.057	<.001	0.864	0.732	0.025	0.657	0.866
215415_s_at	LYST[l]	lysosomal trafficking regulator	–0.395	–0.272	–0.223	–0.101	0.123	<.001	0.104	0.445	0.029	0.458	0.825
203545_at	ALG8	asparagine-linked glycosylation 8, alpha-1,3-glucosyltransferase homolog	–0.390	–0.141	0.01	0.259	0.249	0.002	0.268	0.125	0.048	0.503	0.796

206748_s_at	SPAG9	sperm associated antigen 9	–0.388	–0.150	–0.372	–0.133	0.238	0.002	0.069	0.131	0.044	0.454	0.796
201485_s_at	RCN2	reticulocalbin 2, EF-hand calcium binding domain	–0.381	–0.270	–0.180	–0.069	0.111	<.001	0.373	0.464	0.027	0.53	0.828
212503_s_at	DIP2C	DIP2 disco-interacting protein 2 homolog C	–0.380	–0.350	0.079	0.109	0.029	0.002	0.841	0.883	0.05	0.65	0.885
205880_at	PRKD1	protein kinase D1	–0.380	–0.364	–0.110	–0.095	0.016	0.002	0.218	0.937	0.049	0.487	0.89
219372_at	IFT81	intraflagellar transport 81 homolog	–0.379	–0.217	–0.087	0.076	0.162	0.001	0.615	0.313	0.039	0.595	0.797
212676_at	NF1	neurofibromin 1	–0.374	–0.047	–0.268	0.059	0.326	<.001	0.016	0.012	0.028	0.453	0.796
207515_s_at	POLR1C	polymerase (RNA) I polypeptide C	–0.374	–0.192	–0.280	–0.098	0.182	0.001	0.105	0.211	0.032	0.458	0.797
221759_at	G6PC3	glucose 6 phosphatase, catalytic, 3	–0.373	–0.210	–0.268	–0.105	0.163	<.001	0.087	0.234	0.025	0.454	0.797
213133_s_at	GCSH	similar to Glycine cleavage system H protein	–0.369	–0.225	0.094	0.238	0.144	0.002	0.496	0.374	0.043	0.564	0.809
212797_at	SORT1	sortilin 1	–0.368	–0.030	–0.108	0.231	0.338	0.001	0.012	0.01	0.032	0.453	0.796
209537_at	EXTL2	exostoses (multiple)-like 2	–0.365	–0.339	–0.063	–0.038	0.026	<.001	0.495	0.869	0.025	0.563	0.883
218464_s_at	C17orf63	chromosome 17 open reading frame 63	–0.363	–0.158	–0.251	–0.046	0.205	<.001	0.031	0.108	0.025	0.453	0.796
212996_s_at	URB1	URB1 ribosome biogenesis 1 homolog	–0.357	–0.077	–0.491	–0.211	0.28	0.001	0.008	0.03	0.032	0.453	0.796
213391_at	DPY19L4	dpy-19-like 4	–0.356	–0.335	–0.123	–0.101	0.021	<.001	0.361	0.874	0.025	0.527	0.884
218595_s_at	HEATR1	HEAT repeat containing 1	–0.356	–0.075	–0.278	0.003	0.281	0.002	0.054	0.046	0.045	0.453	0.796
208677_s_at	BSG	basigin (Ok blood group)	–0.352	–0.232	–0.344	–0.225	0.119	0.002	0.164	0.454	0.045	0.468	0.826
210415_s_at	ODF2	outer dense fiber of sperm tails 2	–0.350	–0.100	–0.393	–0.143	0.251	<.001	0.001	0.045	0.029	0.453	0.796
203631_s_at	GPRC5B	G protein-coupled receptor, family C, group 5, member B	–0.349	–0.164	–0.189	–0.005	0.185	<.001	0.207	0.15	0.028	0.483	0.796
201733_at	CLCN3	chloride channel 3	–0.345	–0.257	–0.136	–0.048	0.088	0.002	0.185	0.583	0.044	0.473	0.845
203188_at	B3GNT1	UDP-GlcNAc:betaGal beta-1,3-N-acetylglucosaminyltransferase 1	–0.332	–0.244	–0.059	0.029	0.089	0.002	0.735	0.575	0.048	0.625	0.842
219376_at	ZNF322B	zinc finger protein 322B	–0.329	–0.208	–0.124	–0.003	0.121	<.001	0.178	0.315	0.025	0.472	0.797
200662_s_at	TOMM20	similar to translocase of outer mitochondrial membrane 20 homolog;	–0.328	–0.237	–0.178	–0.087	0.091	<.001	0.345	0.497	0.028	0.523	0.833
218561_s_at	LYRM4	LYR motif containing 4	–0.325	–0.323	–0.206	–0.204	0.001	0.001	0.084	0.994	0.032	0.454	0.893
213854_at	SYNGR1	synaptogyrin 1	–0.324	–0.214	0.079	0.189	0.11	<.001	0.664	0.357	0.025	0.607	0.803

206414_s_at	DDEF2	ArfGAP with SH3 domain, ankyrin repeat and PH domain 2	–0.323	–0.373	–0.077	–0.127	–0.051	0.001	0.342	0.772	0.037	0.522	0.869
218187_s_at	C8orf33	chromosome 8 open reading frame 33	–0.314	–0.206	–0.267	–0.159	0.108	0.002	0.177	0.456	0.048	0.472	0.827
203867_s_at	NLE1	notchless homolog 1	–0.313	–0.135	–0.248	–0.071	0.178	0.001	0.054	0.142	0.034	0.453	0.796
205135_s_at	NUFIP1	nuclear fragile X mental retardation protein interacting protein 1	–0.305	–0.175	–0.163	–0.033	0.13	<.001	0.12	0.252	0.026	0.458	0.797
221532_s_at	WDR61	WD repeat domain 61	–0.305	–0.220	–0.144	–0.058	0.086	0.002	0.118	0.544	0.044	0.458	0.839
212294_at	GNG12	guanine nucleotide binding protein (G protein), gamma 12	–0.302	–0.245	–0.219	–0.163	0.057	0.001	0.389	0.685	0.038	0.535	0.859
211159_s_at	PPP2R5D	protein phosphatase 2, regulatory subunit B', delta isoform	–0.302	–0.051	–0.186	0.064	0.251	0.002	0.024	0.037	0.046	0.453	0.796
212539_at	CHD1L	chromodomain helicase DNA binding protein 1-like	–0.301	–0.232	–0.153	–0.085	0.068	0.001	0.646	0.617	0.038	0.601	0.85
205097_at	SLC26A2	solute carrier family 26 (sulfate transporter), member 2	–0.301	–0.512	0.087	–0.123	–0.210	<.001	0.498	0.287	0.029	0.564	0.797
219880_at	---		–0.297	–0.441	–0.007	–0.151	–0.145	<.001	0.686	0.365	0.025	0.612	0.805
213823_at	HOXA11	homeobox A11	–0.296	–0.028	–0.226	0.042	0.268	0.001	0.04	0.019	0.041	0.453	0.796
200973_s_at	TSPAN3	tetraspanin 3	–0.296	–0.347	0.018	–0.034	–0.051	<.001	0.889	0.718	0.025	0.661	0.865
221586_s_at	E2F5[C]	E2F transcription factor 5, p130-binding	–0.293	–0.001	–0.322	–0.030	0.292	0.002	0.062	0.013	0.048	0.453	0.796
201674_s_at	AKAP1	A kinase (PRKA) anchor protein 1	–0.290	–0.228	0.017	0.08	0.063	0.002	0.855	0.655	0.046	0.655	0.856
221235_s_at	LOC644617	hypothetical LOC644617	–0.290	–0.298	–0.227	–0.235	–0.008	0.002	0.094	0.96	0.044	0.454	0.892
222013_x_at	FAM86B1	family with sequence similarity 86, member B1	–0.283	–0.191	–0.178	–0.087	0.091	0.001	0.247	0.438	0.033	0.496	0.824
204063_s_at	ULK2	unc-51-like kinase 2	–0.283	–0.078	–0.092	0.113	0.205	0.002	0.067	0.072	0.046	0.453	0.796
202749_at	WRB	tryptophan rich basic protein	–0.283	–0.148	–0.083	0.052	0.135	0.001	0.181	0.224	0.033	0.472	0.797
215720_s_at	NFYA	nuclear transcription factor Y, alpha	–0.280	–0.049	–0.275	–0.043	0.232	<.001	0.003	0.014	0.025	0.453	0.796
216986_s_at	IRF4[I]	interferon regulatory factor 4	–0.279	–0.120	–0.133	0.026	0.159	0.002	0.323	0.18	0.049	0.516	0.797
204184_s_at	ADRBK2	adrenergic, beta, receptor kinase 2	–0.275	–0.123	–0.127	0.025	0.152	0.001	0.318	0.17	0.038	0.515	0.796
217935_s_at	UQCC	ubiquinol-cytochrome c reductase complex chaperone	–0.275	–0.127	–0.323	–0.175	0.148	0.001	0.087	0.18	0.038	0.454	0.797
218307_at	RSAD1	radical S-adenosyl methionine domain	–0.274	–0.218	–0.056	<.001	0.056	0.001	0.795	0.649	0.035	0.64	0.853

		containing 1											
221229_s_at	FLJ20628	tRNA methyltransferase 61 homolog B	–0.272	–0.263	–0.124	–0.114	0.009	0.001	0.294	0.945	0.034	0.509	0.892
203284_s_at	HS2ST1	heparan sulfate 2-O-sulfotransferase 1	–0.271	–0.463	0.209	0.017	–0.192	<.001	0.747	0.268	0.027	0.628	0.797
204944_at	PTPRG[C]	protein tyrosine phosphatase, receptor type, G	–0.269	–0.465	–0.089	–0.285	–0.196	0.001	0.333	0.293	0.034	0.519	0.797
213304_at	KIAA0423	family with sequence similarity 179, member B	–0.268	–0.176	–0.078	0.014	0.092	<.001	0.343	0.379	0.028	0.522	0.81
208837_at	TMED3	transmembrane emp24 protein transport domain containing 3	–0.262	–0.416	–0.169	–0.323	–0.154	0.001	0.427	0.376	0.035	0.544	0.809
202019_s_at	LANCL1	LanC lantibiotic synthetase component C-like 1	–0.256	–0.407	–0.032	–0.184	–0.151	0.002	0.074	0.399	0.044	0.454	0.812
208796_s_at	CCNG1	cyclin G1	–0.254	–0.322	–0.013	–0.082	–0.068	0.001	0.393	0.641	0.038	0.537	0.853
200659_s_at	PHB[C]	prohibitin	–0.250	–0.188	–0.147	–0.085	0.062	0.001	0.338	0.567	0.034	0.521	0.841
209777_s_at	SLC19A1	solute carrier family 19 (folate transporter), member 1	–0.250	–0.038	–0.142	0.07	0.213	0.002	0.122	0.033	0.047	0.458	0.796
218709_s_at	IFT52	intraflagellar transport 52 homolog	–0.249	–0.302	–0.142	–0.195	–0.053	0.001	0.21	0.705	0.038	0.483	0.863
211763_s_at	UBE2B	ubiquitin-conjugating enzyme E2B (RAD6 homolog)	–0.249	–0.285	0.047	0.011	–0.036	<.001	0.559	0.775	0.028	0.582	0.869
212745_s_at	BBS4	Bardet-Biedl syndrome 4	–0.245	–0.211	–0.185	–0.151	0.033	0.002	0.096	0.785	0.046	0.455	0.87
213675_at	---		–0.241	–0.405	0.08	–0.084	–0.164	<.001	0.723	0.264	0.025	0.621	0.797
203526_s_at	APC[I,C]	adenomatous polyposis coli	–0.233	–0.185	0.009	0.057	0.048	0.002	0.499	0.665	0.044	0.564	0.857
200972_at	TSPAN3	tetraspanin 3	–0.233	–0.241	0.058	0.05	–0.008	0.001	0.948	0.95	0.039	0.675	0.892
215931_s_at	ARFGEF2	ADP-ribosylation factor guanine nucleotide-exchange factor 2	–0.230	–0.197	–0.026	0.007	0.033	0.002	0.967	0.776	0.049	0.679	0.869
204065_at	CHST10	carbohydrate sulfotransferase 10	–0.229	–0.134	–0.080	0.014	0.094	0.002	0.675	0.36	0.049	0.61	0.803
213508_at	C14orf147	chromosome 14 open reading frame 147	–0.227	–0.550	0.121	–0.202	–0.323	0.002	0.313	0.147	0.045	0.514	0.796
208822_s_at	DAP3	death associated protein 3	–0.227	–0.084	–0.093	0.051	0.143	0.002	0.291	0.131	0.048	0.508	0.796
201380_at	CRTAP	cartilage associated protein	–0.219	–0.400	–0.125	–0.306	–0.181	0.001	0.146	0.269	0.038	0.46	0.797
221488_s_at	CUTA	cutA divalent cation tolerance homolog	–0.219	–0.219	0.017	0.016	<.001	<.001	0.933	0.996	0.029	0.671	0.894
216305_s_at	C2orf3	chromosome 2 open reading frame 3	–0.218	–0.200	–0.189	–0.171	0.018	0.001	0.172	0.862	0.032	0.471	0.882

200929_at	TMED10	transmembrane emp24-like trafficking protein 10	–0.218	–0.217	–0.049	–0.047	0.002	0.001	0.995	0.988	0.038	0.685	0.892
220768_s_at	CSNK1G3	casein kinase 1, gamma 3	–0.215	–0.307	0.035	–0.058	–0.092	0.001	0.634	0.481	0.035	0.599	0.831
205231_s_at	EPM2A	epilepsy, progressive myoclonus type 2A, Lafora disease (laforin)	–0.210	–0.131	–0.165	–0.085	0.08	0.002	0.193	0.395	0.045	0.477	0.811
206961_s_at	MED20	mediator complex subunit 20	–0.206	–0.076	–0.030	0.101	0.131	<.001	0.162	0.078	0.028	0.466	0.796
208716_s_at	TMCO1	transmembrane and coiled-coil domains 1	–0.205	–0.114	0.054	0.145	0.091	0.002	0.428	0.309	0.046	0.544	0.797
202300_at	HBXIP	hepatitis B virus x interacting protein	–0.204	–0.133	–0.009	0.062	0.071	0.001	0.549	0.42	0.038	0.58	0.82
205217_at	TIMM8A	translocase of inner mitochondrial membrane 8 homolog A	–0.204	–0.048	–0.095	0.061	0.156	0.002	0.108	0.056	0.045	0.458	0.796
212122_at	RHOQ, RHOQP2	ras homolog gene family, member Q	–0.201	–0.233	0.113	0.081	–0.032	0.002	0.886	0.783	0.047	0.66	0.869
218437_s_at	LZTFL1	leucine zipper transcription factor-like 1	–0.199	–0.305	–0.083	–0.188	–0.105	<.001	0.6	0.377	0.028	0.592	0.809
201059_at	CTTN[c]	cortactin	–0.195	–0.189	–0.073	–0.067	0.006	0.001	0.253	0.953	0.039	0.498	0.892
203733_at	DEXI	dexamethasone-induced transcript	–0.194	–0.316	0.049	–0.073	–0.122	0.002	0.763	0.392	0.049	0.632	0.81
214052_x_at	BAT2D1	proline-rich coiled-coil 2C	–0.192	–0.064	–0.121	0.007	0.128	<.001	0.147	0.065	0.029	0.46	0.796
202033_s_at	RB1CC1	RB1-inducible coiled-coil 1	–0.192	–0.288	–0.119	–0.215	–0.096	0.002	0.322	0.455	0.043	0.516	0.827
211976_at	RPL35P2, RPL35P1, rpl35	ribosomal protein L35; ribosomal protein L35 pseudogene 1; ribosomal protein L35 pseudogene 2	–0.191	–0.289	–0.146	–0.245	–0.098	0.001	0.211	0.441	0.041	0.484	0.824
214877_at	CDKAL1	CDK5 regulatory subunit associated protein 1-like 1	–0.187	–0.161	–0.199	–0.173	0.025	<.001	0.362	0.747	0.025	0.527	0.867
211094_s_at	NF1	nuclear transcription factor Y, alpha	–0.181	–0.140	–0.061	–0.020	0.041	0.002	0.32	0.636	0.046	0.516	0.853
212987_at	FBXO9	F-box protein 9	–0.176	–0.246	–0.028	–0.097	–0.070	0.002	0.433	0.54	0.046	0.546	0.838
202371_at	TCEAL4	transcription elongation factor A (SII)-like 4	–0.176	–0.313	0.251	0.114	–0.137	<.001	0.488	0.246	0.028	0.561	0.797
208251_at	KCNC4	potassium voltage-gated channel, Shaw-related subfamily, member 4	–0.169	–0.040	–0.126	0.003	0.129	0.002	0.157	0.06	0.049	0.464	0.796
202034_x_at	RB1CC1	RB1-inducible coiled-coil 1	–0.167	–0.306	–0.045	–0.184	–0.139	<.001	0.252	0.236	0.03	0.498	0.797

204823_at	NAV3	neuron navigator 3; similar to neuron navigator 3	–0.165	–0.537	0.67	0.298	–0.372	0.001	0.018	0.071	0.039	0.453	0.796
214462_at	SOCS6	suppressor of cytokine signaling 6	–0.161	–0.150	–0.040	–0.029	0.011	0.001	0.151	0.881	0.034	0.462	0.885
210974_s_at	AP3D1[I]	adaptor-related protein complex 3, delta 1 subunit	–0.159	–0.123	–0.081	–0.046	0.035	0.002	0.348	0.64	0.045	0.524	0.853
218203_at	ALG5	asparagine-linked glycosylation 5, dolichyl-phosphate beta-glucosyltransferase homolog	–0.158	–0.209	0.219	0.168	–0.051	0.001	0.272	0.593	0.039	0.504	0.846
203866_at	NLE1	notchless homolog 1	–0.158	–0.063	–0.100	–0.004	0.095	0.002	0.251	0.146	0.046	0.497	0.796
212229_s_at	FBXO21	F-box protein 21	–0.155	–0.307	–0.069	–0.220	–0.151	0.001	0.181	0.219	0.038	0.472	0.797
204498_s_at	ADCY9	adenylate cyclase 9	–0.154	–0.067	–0.061	0.027	0.087	0.002	0.153	0.178	0.046	0.463	0.797
212505_s_at	KIAA0892	MAU2 chromatid cohesion factor homolog	–0.150	–0.102	–0.153	–0.105	0.048	0.002	0.107	0.484	0.045	0.458	0.831
202440_s_at	ST5[C]	suppression of tumorigenicity 5	–0.147	–0.354	–0.040	–0.248	–0.207	0.001	0.022	0.134	0.038	0.453	0.796
200719_at	SKP1	S-phase kinase-associated protein 1	–0.139	–0.380	–0.001	–0.242	–0.241	0.001	0.087	0.102	0.039	0.454	0.796
212169_at	FKBP9	FK506 binding protein 9, 63 kDa	–0.138	–0.439	–0.204	–0.505	–0.301	0.002	0.063	0.081	0.044	0.453	0.796
221279_at	GDAP1	ganglioside-induced differentiation-associated protein 1	–0.127	–0.191	0.024	–0.040	–0.064	<.001	0.565	0.349	0.025	0.584	0.801
200983_x_at	CD59	CD59 molecule, complement regulatory protein	–0.111	–0.293	0.168	–0.014	–0.183	0.001	0.437	0.114	0.041	0.546	0.796
200984_s_at	CD59	CD59 molecule, complement regulatory protein	–0.090	–0.378	0.219	–0.069	–0.288	0.002	0.253	0.048	0.044	0.498	0.796
202853_s_at	RYK	RYK receptor-like tyrosine kinase	–0.090	–0.224	0.046	–0.088	–0.134	0.002	0.478	0.143	0.046	0.558	0.796
210844_x_at	CTNNA1	catenin (cadherin-associated protein), alpha 1, 102kDa	–0.085	–0.211	–0.027	–0.154	–0.127	0.001	0.064	0.111	0.034	0.453	0.796
208407_s_at	CTNND1	catenin (cadherin-associated protein), delta 1	–0.082	–0.423	0.164	–0.177	–0.341	0.002	0.223	0.04	0.048	0.487	0.796
219078_at	GPATCH2	G patch domain containing 2	–0.081	–0.325	0.025	–0.219	–0.244	<.001	0.116	0.032	0.03	0.458	0.796
203141_s_at	AP3B1[I]	adaptor-related protein complex 3, beta 1 subunit	–0.069	–0.215	0.058	–0.088	–0.146	<.001	0.318	0.049	0.028	0.515	0.796
200811_at	CIRBP	cold inducible RNA	–0.068	–0.423	0.035	–0.320	–0.355	<.001	0.023	0.01	0.025	0.453	0.796

		binding protein											
200711_s_at	SKP1	S-phase kinase-associated protein 1	–0.063	–0.233	0.058	–0.112	–0.170	0.001	0.061	0.042	0.034	0.453	0.796
200985_s_at	CD59[I]	CD59 molecule, complement regulatory protein	–0.062	–0.381	0.215	–0.105	–0.320	<.001	0.066	0.008	0.025	0.453	0.796
213040_s_at	NPTXR	neuronal pentraxin receptor	–0.020	–0.322	0.175	–0.127	–0.302	<.001	0.017	0.006	0.029	0.453	0.796
212417_at	SCAMP1	secretory carrier membrane protein 1	–0.015	–0.368	0.169	–0.184	–0.354	<.001	0.055	0.005	0.028	0.453	0.796
208733_at	RAB2A	RAB2A, member RAS oncogene family	–0.013	–0.180	0.201	0.034	–0.167	0.002	0.019	0.018	0.05	0.453	0.796
200810_s_at	CIRBP	cold inducible RNA binding protein	–0.008	–0.376	0.031	–0.337	–0.368	<.001	0.006	0.003	0.025	0.453	0.796

I = immune related; C = cancer related; M = melanoma related
