# Supplementary material for: A prospective phase II trial exploring the association between tumor microenvironment biomarkers and clinical activity of ipilimumab in advanced melanoma
Source: J Transl Med. 2011 Nov 28;9:204. doi: 10.1186/1479-5876-9-204 (PMC3239318; doi:10.1186/1479-5876-9-204)
Supplement: Additional file 9 — Table S8. Model estimates for probe sets with time effect q-value < 0.05 and opposite direction of change from baseline. [file 1479-5876-9-204-S9.PDF]

Table S8 Model estimates for probe sets with time effect q-value <0.05 and opposite direction of change from baseline.

Probe Set ID	Gene Symbol	Gene Name	Time Effect 3 mg/kg	Time Effect 10 mg/kg	Dose Effect Pre-Tx	Dose Effect Post-Tx	Interaction Effect	Time Effect *P*-value	Dose Effect *P*-value	Interaction *P*-value	Time Effect q-value	Dose Effect q-value	Interaction q-value
211020_at	GCNT2	glucosaminyl (N-acetyl) transferase 2, I-branching enzyme (I blood group)	–0.554	0.023	–0.630	–0.053	0.577	0.002	0.001	0.01	0.049	0.453	0.796
204108_at	NFYA	nuclear transcription factor Y, alpha	–0.443	0.043	–0.449	0.037	0.486	<.001	<.001	<.001	0.025	0.453	0.796
209870_s_at	APBA2	amyloid beta (A4) precursor protein-binding, family A, member 2	–0.318	0.064	–0.270	0.113	0.383	0.002	0.018	0.003	0.045	0.453	0.796
218724_s_at	TGIF2[C]	TGFB-induced factor homeobox 2	–0.288	0.002	–0.260	0.03	0.29	<.001	0.001	0.003	0.027	0.453	0.796
202895_s_at	SIRPA[I]	signal-regulatory protein alpha (immunoglobulin)	–0.265	0.09	–0.351	0.004	0.355	0.001	0.002	0.001	0.035	0.453	0.796
213763_at	HIPK2	homeodomain interacting protein kinase 2	–0.244	0.22	–0.418	0.046	0.464	0.002	0.002	<.001	0.047	0.453	0.796
221997_s_at	MRPL52	mitochondrial ribosomal protein L52	–0.192	0.001	–0.178	0.016	0.194	0.002	0.014	0.01	0.044	0.453	0.796
218060_s_at	C16orf57	chromosome 16 open reading frame 57	–0.025	0.228	–0.179	0.074	0.253	0.001	0.021	0.003	0.036	0.453	0.796
208876_s_at	PAK2	p21 protein (Cdc42/Rac)-activated kinase 2	–0.015	0.222	–0.078	0.16	0.238	0.001	0.006	0.003	0.035	0.453	0.796
212416_at	SCAMP1	secretory carrier membrane protein 1	0.075	–0.329	0.26	–0.144	–0.404	0.001	0.047	0.002	0.039	0.453	0.796
204840_s_at	EEA1	early endosome antigen 1	0.108	–0.128	0.073	–0.163	–0.236	0.001	0.001	<.001	0.041	0.453	0.796
203324_s_at	CAV2[C]	caveolin 2	0.121	–0.470	0.323	–0.268	–0.592	<.001	0.003	0.001	0.03	0.453	0.796
212706_at	RASA4	RAS p21 protein activator 4	0.297	–0.098	0.12	–0.276	–0.396	0.001	0.004	0.001	0.039	0.453	0.796
203232_s_at	ATXN1	ataxin 1	0.399	–0.211	0.316	–0.294	–0.610	0.002	0.009	0.001	0.046	0.453	0.796
208335_s_at	DARC[I]	Duffy blood group, chemokine receptor	0.962	–0.316	0.646	–0.631	–1.278	0.002	0.017	0.002	0.046	0.453	0.796

I = immune related; C = cancer related
